# On Pixel-Wise Explanations for Non-Linear Classifier Decisions by Layer-Wise Relevance Propagation

**DOI:** 10.1371/journal.pone.0130140

**Published:** 2015-07-10

**Authors:** Sebastian Bach, Alexander Binder, Grégoire Montavon, Frederick Klauschen, Klaus-Robert Müller, Wojciech Samek

**Affiliations:** 1 Machine Learning Group, Fraunhofer Heinrich Hertz Institute, Berlin, Germany; 2 Machine Learning Group, Technische Universität Berlin, Berlin, Germany; 3 Charité University Hospital, Berlin, Germany; 4 Department of Brain and Cognitive Engineering, Korea University, Seoul, Korea; 5 ISTD Pillar, Singapore University of Technology and Design (SUTD), Singapore; Universidad de Castilla-La Mancha, SPAIN

## Abstract

Understanding and interpreting classification decisions of automated image classification systems is of high value in many applications, as it allows to verify the reasoning of the system and provides additional information to the human expert. Although machine learning methods are solving very successfully a plethora of tasks, they have in most cases the disadvantage of acting as a black box, not providing any information about what made them arrive at a particular decision. This work proposes a general solution to the problem of understanding classification decisions by pixel-wise decomposition of nonlinear classifiers. We introduce a methodology that allows to visualize the contributions of single pixels to predictions for kernel-based classifiers over Bag of Words features and for multilayered neural networks. These pixel contributions can be visualized as heatmaps and are provided to a human expert who can intuitively not only verify the validity of the classification decision, but also focus further analysis on regions of potential interest. We evaluate our method for classifiers trained on PASCAL VOC 2009 images, synthetic image data containing geometric shapes, the MNIST handwritten digits data set and for the pre-trained ImageNet model available as part of the Caffe open source package.

## Introduction

Classification of images has become a key ingredient in many computer vision applications, e.g. image search [1], robotics [2], medical imaging [3], object detection in radar images [4] or face detection [5]. Two particularly popular approaches, neural networks [6] and Bag of Words (BoW) models [7], are widely used for these tasks and were among the top submissions in competitions on image classification and ranking such as ImageNet [8], Pascal VOC [9] and ImageCLEF [10]. However, like many methods in machine learning, these models often lack a straightforward interpretability of the classifier predictions. In other words the classifier acts as a *black box* and does not provide detailed information about why it reaches a certain classification decision.

This lack of interpretability is due to the non-linearity of the various mappings that process the raw image pixels to its feature representation and from that to the final classifier function. This is a considerable drawback in classification applications, as it hinders the human experts to carefully verify the classification decision. A simple *yes* or *no* answer is sometimes of limited value in applications, where questions like *where* something occurs or *how* it is structured are more relevant than a binary or real-valued one-dimensional assessment of mere presence or absence of a certain structure.

In this work, we aim to close the gap between classification and interpretability both for multilayered neural networks and Bag of Words (BoW) models over non-linear kernels which are two classes of predictors which enjoy popularity in computer vision. We will consider both types of predictors in a generic sense trying to avoid whenever possible a priori restrictions to specific algorithms or mappings. For the first part, the Bag of Words models will be treated as an aggregation of non-linear mappings over local features in an image which includes a number of popular mapping methods such as Fisher vectors [11], regularized coding [12–14] and soft coding [15], combined with differentiable non-linear kernels and a range of pooling functions including sum- and max-pooling. For the second part, the neural networks (e.g. [6, 16]), we will consider general multilayered network structures with arbitrary continuous neurons and pooling functions based on generalized p-means.

The next Section *Pixel-wise Decomposition as a General Concept* will explain the basic approaches underlying the pixel-wise decomposition of classifiers. In Section *Bag of Words models revisited*, we will give a short recapitulation about Bag of Words features and kernel-based classifiers and summarize related work. *Overview of the decomposition steps* will discuss the decomposition of a kernel-based classifier into sums of scores over small regions of the image, and the projection down to single pixels. Our method then is applied to a number of popular mappings and kernels in Section *Examples for various mappings and kernels*. *Pixel-wise Decomposition for Multilayer Networks* applies both the Taylor-based and layer-wise relevance propagation approaches explained in *Pixel-wise Decomposition as a General Concept* to neural network architectures. The experimental evaluation of our framework will be done in *Experiments* and we conclude the paper with a discussion in *Discussion*.

## Pixel-wise Decomposition as a General Concept

The overall idea of pixel-wise decomposition is to understand the contribution of a single pixel of an image *x* to the prediction *f*(*x*) made by a classifier *f* in an image classification task. We would like to find out, separately for each image *x*, which pixels contribute to what extent to a positive or negative classification result. Furthermore we want to express this extent quantitatively by a measure. We assume that the classifier has real-valued outputs which are thresholded at zero. In such a setup it is a mapping *f* : ℝ^*V*^ → ℝ^1^ such that *f*(*x*) > 0 denotes presence of the learned structure. Probabilistic outputs can be treated without loss of generality by subtracting 0.5. We are interested to find out the contribution of each input pixel *x*_(*d*)_ of an input image *x* to a particular prediction *f*(*x*). The important constraint specific to classification consists in finding the differential contribution relative to the state of maximal uncertainty with respect to classification which is then represented by the set of root points *f*(*x*_0_) = 0. One possible way is to decompose the prediction *f*(*x*) as a sum of terms of the separate input dimensions *x*_*d*_ respectively pixels:

$$\begin{array}{c} f\left(x\right)\approx \sum_{d=1}^{V}R_{d} \end{array}$$ (1)

**Fig 1:**
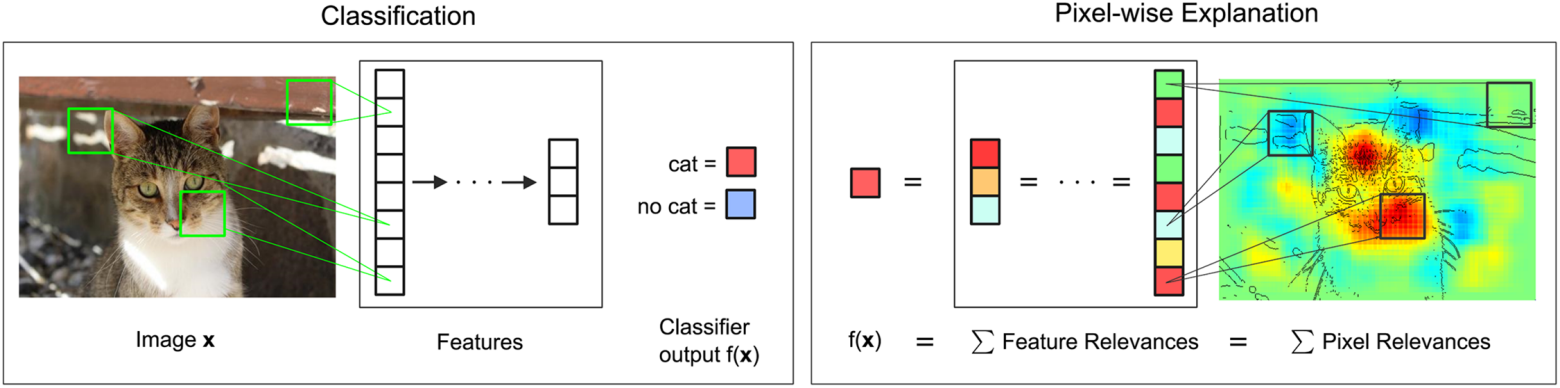
Visualization of the pixel-wise decomposition process. In the classification step the image is converted to a feature vector representation and a classifier is applied to assign the image to a given category, e.g., “cat” or “no cat”. Note that the computation of the feature vector usually involves the usage of several intermediate representations. Our method decomposes the classification output *f*(*x*) into sums of feature and pixel relevance scores. The final relevances visualize the contributions of single pixels to the prediction. Cat image by pixabay user stinne24.

The qualitative interpretation is that *R*_*d*_ < 0 contributes evidence against the presence of a structure which is to be classified while *R*_*d*_ > 0 contributes evidence for its presence. In terms of subsequent visualization, which however will not be the scope of this paper, the resulting relevances *R*_*d*_ for each input pixel *x*_(*d*)_ can be mapped to a color space and visualized in that way as a conventional heatmap. One basic constraint will be in the following work that the signs of *R*_*d*_ should follow above qualitative interpretation, i.e. positive values should denote positive contributions, negative values negative contributions. Fig 1 depicts the main idea of our method.

In this paper we propose a novel concept we denote as *layer-wise relevance propagation* as a general concept for the purpose of achieving a pixel-wise decomposition as in Eq (1). We also discuss an approach based on *Taylor decomposition* which yields an approximation of layer-wise relevance propagation. We will show that for a wide range of non-linear classification architectures, layer-wise relevance propagation can be done without the need to use an approximation by means of Taylor expansion. The methods we propose in this paper do not involve segmentation. They do not require pixel-wise training as learning setup or pixel-wise labeling for the training phase. The setup used here is image-wise classification, in which during training one label is provided for an image as a whole, however, the contribution of this paper is not about classifier training. The proposed methods are built on top of a pre-trained classifier. They are even applicable to an already pre-trained image classifier as shown in Section *Neural Network for 1000 ILSVRC classes*.

### Layer-wise relevance propagation

We will introduce layer-wise relevance propagation as a concept defined by a set of constraints. Any solution satisfying the constraints will be considered to follow the concept of layer-wise relevance propagation. In later sections we will then derive solutions for two particular classifier architectures and evaluate these solutions experimentally for their meaningfulness. Layer-wise relevance propagation in its general form assumes that the classifier can be decomposed into several layers of computation. Such layers can be parts of the feature extraction from the image or parts of a classification algorithm run on the computed features. As shown later, this is possible for Bag of Words features with non-linear SVMs as well as for neural networks.

The first layer are the inputs, the pixels of the image, the last layer is the real-valued prediction output of the classifier *f*. The *l*-th layer is modeled as a vector $z={\left(z_{d}^{\left(l\right)}\right)}_{d=1}^{V(l)}$ with dimensionality *V*(*l*). Layer-wise relevance propagation assumes that we have a Relevance score $R_{d}^{\left(l+1\right)}$ for each dimension $z_{d}^{\left(l+1\right)}$ of the vector *z* at layer *l* + 1. The idea is to find a Relevance score $R_{d}^{\left(l\right)}$ for each dimension $z_{d}^{\left(l\right)}$ of the vector *z* at the next layer *l* which is closer to the input layer such that the following equation holds.

$$\begin{array}{c} f\left(x\right)=\cdots =\sum_{d\in l+1}R_{d}^{\left(l+1\right)}=\sum_{d\in l}R_{d}^{\left(l\right)}=\cdots =\sum_{d}R_{d}^{\left(1\right)} \end{array}$$ (2)

Iterating Eq (2) from the last layer which is the classifier output *f*(*x*) down to the input layer *x* consisting of image pixels then yields the desired Eq (1). The Relevance for the input layer will serve as the desired sum decomposition in Eq (1). In the following we will derive further constraints beyond Eqs (1) and (2) and motivate them by examples. As we will show now, a decomposition satisfying Eq (2) per se is neither unique, nor it is guaranteed that it yields a meaningful interpretation of the classifier prediction.

We give here a simple counterexample. Suppose we have one layer. The inputs are *x* ∈ ℝ^*V*^. We use a linear classifier with some arbitrary and dimension-specific feature space mapping *ϕ*_*d*_ and a bias *b*

$$\begin{array}{c} f\left(x\right)=b+\sum_{d}\alpha_{d}\phi_{d}\left(x_{d}\right) \end{array}$$ (3)

Let us define the relevance for the second layer trivially as $R_{1}^{\left(2\right)}=f(x)$. Then, one possible layer-wise relevance propagation formula would be to define the relevance *R* ^(1)^ for the inputs *x* as

$$\begin{array}{ccc} R_{d}^{\left(1\right)} & = & \left\{ \begin{array}{cc} f\left(x\right)\frac{\left|\alpha_{d}\phi_{d}\left(x_{d}\right)\right|}{\sum_{d}|\alpha_{d}\phi_{d}\left(x_{d}\right)|} & \text{if}\;\sum_{d}|\alpha_{d}\phi_{d}\left(x_{d}\right)|\neq 0\\ \frac{b}{V} & \text{if}\;\sum_{d}|\alpha_{d}\phi_{d}\left(x_{d}\right)|=0 \end{array}\right. \end{array}$$ (4)

This clearly satisfies Eqs (1) and (2), however the Relevances *R* ^(1)^(*x*_*d*_) of all input dimensions have the same sign as the prediction *f*(*x*). In terms of pixel-wise decomposition interpretation, all inputs point towards the presence of a structure if *f*(*x*) > 0 and towards the absence of a structure if *f*(*x*) < 0. This is for many classification problems not a realistic interpretation.

Let us discuss a more meaningful way of defining layer-wise relevance propagation. For this example we define

$$\begin{array}{c} R_{d}^{\left(1\right)}=\frac{b}{V}+\alpha_{d}\phi_{d}\left(x_{d}\right) \end{array}$$ (5)

Then, the relevance of a feature dimension *x*_*d*_ depends on the sign of the term in Eq (5). This is for many classification problems a more plausible interpretation. This second example shows that the layer-wise relevance propagation is able to deal with non-linearities such as the feature space mapping *ϕ*_*d*_ to some extent and how an example of layer-wise relevance propagation satisfying Formula (2) may look like in practice. Note that no regularity assumption on the feature space mapping *ϕ*_*d*_ is required here at all, it could be even non-continuous, or non-measurable under the Lebesgue measure. The underlying Formula (2) can be interpreted as a conservation law for the relevance *R* in between layers of the feature processing.

The above example gives furthermore an intuition about what relevance *R* is, namely, the local contribution to the prediction function *f*(*x*). In that sense the relevance of the output layer is the prediction itself *f*(*x*). This first example shows what one could expect as a decomposition for the linear case. The linear case is not a novelty, however, it provides a first intuition.

**Fig 2:**
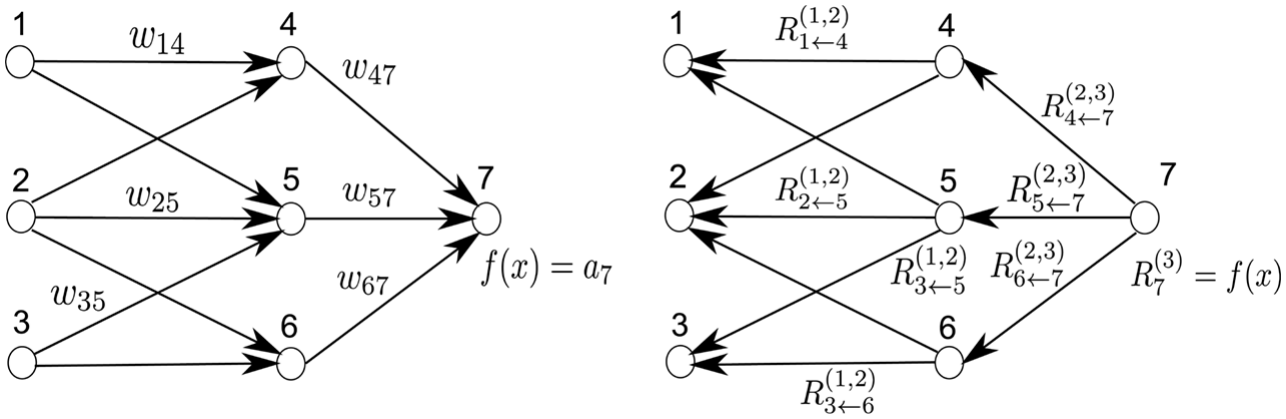
Left: A neural network-shaped classifier during prediction time. *w*_*ij*_ are connection weights. *a*_*i*_ is the activation of neuron *i*. Right: The neural network-shaped classifier during layer-wise relevance computation time. $R_{i}^{\left(l\right)}$ is the relevance of neuron *i* which is to be computed. In order to facilitate the computation of $R_{i}^{\left(l\right)}$ we introduce messages $R_{i\leftarrow j}^{\left(l,l+1\right)}$. $R_{i\leftarrow j}^{\left(l,l+1\right)}$ are messages which need to be computed such that the layer-wise relevance in Eq (2) is conserved. The messages are sent from a neuron *i* to its input neurons *j* via the connections used for classification, e.g. 2 is an input neuron for neurons 4, 5, 6. Neuron 3 is an input neuron for 5, 6. Neurons 4, 5, 6 are the input for neuron 7.

We give a second, more graphic and non-linear, example. The left panel of Fig 2 shows a neural network-shaped classifier with neurons and weights *w*_*ij*_ on connections between neurons. Each neuron *i* has an output *a*_*i*_ from an activation function.

The top layer consists of one output neuron, indexed by 7. For each neuron *i* we would like to compute a relevance *R*_*i*_. We initialize the top layer relevance $R_{7}^{\left(3\right)}$ as the function value, thus $R_{7}^{\left(3\right)}=f(x)$. Layer-wise relevance propagation in Eq (2) requires now to hold

$$\begin{array}{c} R_{7}^{\left(3\right)}=R_{4}^{\left(2\right)}+R_{5}^{\left(2\right)}+R_{6}^{\left(2\right)} \end{array}$$ (6)

$$\begin{array}{c} R_{4}^{\left(2\right)}+R_{5}^{\left(2\right)}+R_{6}^{\left(2\right)}=R_{1}^{\left(1\right)}+R_{2}^{\left(1\right)}+R_{3}^{\left(1\right)} \end{array}$$ (7)

We will make two assumptions for this example. Firstly, we express the layer-wise relevance in terms of messages $R_{i\leftarrow j}^{\left(l,l+1\right)}$ between neurons *i* and *j* which can be sent along each connection. The messages are, however, directed from a neuron towards its input neurons, in contrast to what happens at prediction time, as shown in the right panel of Fig 2. Secondly, we define the relevance of any neuron except neuron 7 as the sum of incoming messages:

$$\begin{array}{c} R_{i}^{\left(l\right)}=\sum_{k:\;i\;\text{is}\;\text{input}\;\text{for}\;\text{neuron}\;k}R_{i\leftarrow k}^{\left(l,l+1\right)} \end{array}$$ (8)

For example $R_{3}^{\left(1\right)}=R_{3\leftarrow 5}^{\left(1,2\right)}+R_{3\leftarrow 6}^{\left(1,2\right)}$. Note that neuron 7 has no incoming messages anyway. Instead its relevance is defined as $R_{7}^{\left(3\right)}=f(x)$. In Eq (8) and the following text the terms *input* and *source* have the meaning of being an input to another neuron in the direction as defined during classification time, not during the time of computation of layer-wise relevance propagation. For example in Fig 2 neurons 1 and 2 are inputs and source for neuron 4, while neuron 6 is the *sink* for neurons 2 and 3. Given the two assumptions encoded in Eq (8), the layer-wise relevance propagation by Eq (2) can be satisfied by the following sufficient condition:

$$\begin{array}{c} R_{7}^{\left(3\right)}=R_{4\leftarrow 7}^{\left(2,3\right)}+R_{5\leftarrow 7}^{\left(2,3\right)}+R_{6\leftarrow 7}^{\left(2,3\right)} \end{array}$$ (9)

$$\begin{array}{c} R_{4}^{\left(2\right)}=R_{1\leftarrow 4}^{\left(1,2\right)}+R_{2\leftarrow 4}^{\left(1,2\right)} \end{array}$$ (10)

$$\begin{array}{c} R_{5}^{\left(2\right)}=R_{1\leftarrow 5}^{\left(1,2\right)}+R_{2\leftarrow 5}^{\left(1,2\right)}+R_{3\leftarrow 5}^{\left(1,2\right)} \end{array}$$ (11)

$$\begin{array}{c} R_{6}^{\left(2\right)}=R_{2\leftarrow 6}^{\left(1,2\right)}+R_{3\leftarrow 6}^{\left(1,2\right)} \end{array}$$ (12)

In general, this condition can be expressed as:

$$\begin{array}{c} R_{k}^{\left(l+1\right)}=\sum_{i:\;i\;\text{is}\;\text{input}\;\text{for}\;\text{neuron}\;k}R_{i\leftarrow k}^{\left(l,l+1\right)} \end{array}$$ (13)

The difference between condition (13) and definition (8) is that in the condition (13) the sum runs over the sources at layer *l* for a fixed neuron *k* at layer *l*+1, while in the definition (8) the sum runs over the sinks at layer *l*+1 for a fixed neuron *i* at a layer *l*. When using Eq (8) to define the relevance of a neuron from its messages, then condition (13) is a sufficient condition in order to ensure that Eq (2) holds. Summing over the left hand side in Eq (13) yields

$$\begin{array}{ccc} \sum_{k}R_{k}^{\left(l+1\right)} & = & \sum_{k}\sum_{i:\;i\;\text{is}\;\text{input}\;\text{for}\;\text{neuron}\;k}R_{i\leftarrow k}^{\left(l,l+1\right)}\\ & = & \sum_{i}\sum_{k:\;i\;\text{is}\;\text{input}\;\text{for}\;\text{neuron}\;k}R_{i\leftarrow k}^{\left(l,l+1\right)}\\ & \overset{eq.(8)}{=} & \sum_{i}R_{i}^{\left(l\right)} \end{array}$$

One can interpret condition (13) by saying that the messages $R_{i\leftarrow k}^{\left(l,l+1\right)}$ are used to distribute the relevance $R_{k}^{\left(l+1\right)}$ of a neuron *k* onto its input neurons at layer *l*. Our work in the following sections will be based on this notion and the more strict form of relevance conservation as given by definition (8) and condition (13). We set Eqs (8) and (13) as the main constraints defining layer-wise relevance propagation. A solution following this concept is required to define the messages $R_{i\leftarrow k}^{\left(l,l+1\right)}$ according to these equations.

Now we can derive an explicit formula for layer-wise relevance propagation for our example by defining the messages $R_{i\leftarrow k}^{\left(l,l+1\right)}$. The layer-wise relevance propagation should reflect the messages passed during classification time. We know that during classification time, a neuron *i* inputs *a*_*i*_ *w*_*ik*_ to neuron *k*, provided that *i* has a forward connection to *k*. Thus, we can rewrite the left hand sides of Eqs (9) and (10) so that it matches the structure of the right hand sides of the same equations by the following

$$\begin{array}{c} R_{7}^{\left(3\right)}=R_{7}^{\left(3\right)}\frac{a_{4}w_{47}}{\sum_{i=4,5,6}a_{i}w_{i7}}+R_{7}^{\left(3\right)}\frac{a_{5}w_{57}}{\sum_{i=4,5,6}a_{i}w_{i7}}+R_{7}^{\left(3\right)}\frac{a_{6}w_{67}}{\sum_{i=4,5,6}a_{i}w_{i7}} \end{array}$$ (14)

$$\begin{array}{c} R_{4}^{\left(2\right)}=R_{4}^{\left(2\right)}\frac{a_{1}w_{14}}{\sum_{i=1,2}a_{i}w_{i4}}+R_{4}^{\left(2\right)}\frac{a_{2}w_{24}}{\sum_{i=1,2}a_{i}w_{i4}} \end{array}$$ (15)

The match of the right hand sides of Eqs (9) and (10) against the right hand sides of (14) and (15) can be expressed in general as

$$\begin{array}{c} R_{i\leftarrow k}^{\left(l,l+1\right)}=R_{k}^{\left(l+1\right)}\frac{a_{i}w_{ik}}{\sum_{h}a_{h}w_{hk}} \end{array}$$ (16)

While this solution (16) for message terms $R_{i\leftarrow k}^{\left(l,l+1\right)}$ still needs to be adapted such that it is usable when the denominator becomes zero, the example given in Eq (16) gives an idea what a message $R_{i\leftarrow k}^{\left(l,l+1\right)}$ could be, namely the relevance of a sink neuron $R_{k}^{\left(l+1\right)}$ which has been already computed, weighted proportionally by the input of the neuron *i* from the preceding layer *l*. This notion holds in an analogous way when we use different classification architectures and replace the notion of a neuron by a dimension of a feature vector at a given layer.

The Formula (16) has a second property: The sign of the relevance sent by message $R_{i\leftarrow k}^{\left(l,l+1\right)}$ becomes inverted if the contribution of a neuron *a*_*i*_ *w*_*ik*_ has different sign then the sum of the contributions from all input neurons, i.e. if the neuron fires against the overall trend for the top neuron from which it inherits a portion of the relevance. Same as for the example with the linear mapping in Eq (5), an input neuron can inherit positive or negative relevance depending on its input sign. This is a difference to the Eq (4). While this sign switching property can be defined analogously for a range of architectures, we do not add it as a constraint for layer-wise relevance propagation.

One further property is visible here as well. The formula for distribution of relevance is applicable to non-linear and even non-differentiable or non-continuous neuron activations *a*_*k*_. An algorithm would start with relevances *R* ^(*l*+1)^ of layer *l*+1 which have been computed already. Then the messages $R_{i\leftarrow k}^{\left(l,l+1\right)}$ would be computed for all elements *k* from layer *l*+1 and elements *i* from the preceding layer *l*—in a manner such that Eq (13) holds. Then definition (8) would be used to define the relevances *R* ^(*l*)^ for all elements of layer *l*.

The relevance conservation property can in principle be supplemented by other constraints that further reduce the set of admissible solutions. For example, one could constrain relevance messages $R_{i\leftarrow k}^{\left(l,l+1\right)}$ that result from the redistribution of relevance onto lower-level nodes in a way that is consistent with the contribution of lower-level nodes to the upper layer during the forward pass. If a node *i* has a larger weighted activation *z*_*ik*_ = *a*_*i*_ *w*_*ik*_, then, in a qualitative sense, it should also receive a larger fraction of the relevance score $R_{k}^{\left(l+1\right)}$ of the node *k*. In particular, for all nodes *k* satisfying *R*_*k*_, ∑_*i*_ *z*_*ik*_ > 0, one can define the constraint $0<z_{ik}<z_{i^{\prime }k}\Rightarrow R_{i\leftarrow k}^{\left(l,l+1\right)}\leq R_{i^{\prime }\leftarrow k}^{\left(l,l+1\right)}$. (The formulas provided in section *Pixel-wise Decomposition for Multilayer Networks* adhere to this ordering constraint.) Nevertheless, nothing is said about the exact relevance values beyond their adherence to the constraint stated above.

To summarize, we have introduced layer-wise relevance propagation in a feed-forward network. In our proposed definition, the total relevance is constrained to be preserved from one layer to another, and the total node relevance must be the equal to the sum of all relevance messages incoming to this node and also equal to the sum of all relevance messages that are outgoing to the same node. It is important to note that the definition is *not* given as an algorithm or a solution of an objective with a distinct minimum. Instead, it is given as a set of constraints that the solution should satisfy. Thus, different algorithms with different resulting solutions may be admissible under these constraints.

### Taylor-type decomposition

One alternative approach for achieving a decomposition as in (1) for a general differentiable predictor *f* is first order Taylor approximation.

$$\begin{array}{ccc} f(x) & \approx & f\left(x_{0}\right)+Df\left(x_{0}\right)\left[x-x_{0}\right]\\ & = & f\left(x_{0}\right)+\sum_{d=1}^{V}\frac{\partial f}{\partial x_{\left(d\right)}}\left(x_{0}\right)\left(x_{\left(d\right)}-x_{0(d)}\right) \end{array}$$ (17)

The choice of a Taylor base point *x*_0_ is a free parameter in this setup. As said above, in case of classification we are interested to find out the contribution of each pixel relative to the state of maximal uncertainty of the prediction which is given by the set of points *f*(*x*_0_) = 0, since *f*(*x*) > 0 denotes presence and *f*(*x*) < 0 absence of the learned structure. Thus, *x*_0_ should be chosen to be a root of the predictor *f*. For the sake of precision of the Taylor approximation of the prediction, *x*_0_ should be chosen to be close to *x* under the Euclidean norm in order to minimize the Taylor residuum according to higher order Taylor approximations. In case of multiple existing roots *x*_0_ with minimal norm, they can be averaged or integrated in order to get an average over all these solutions. The above equation simplifies to

$$\begin{array}{c} f\left(x\right)\approx \sum_{d=1}^{V}\frac{\partial f}{\partial x_{\left(d\right)}}\left(x_{0}\right)\left(x_{\left(d\right)}-x_{0(d)}\right)\;\;\text{such}\;\text{that}\;f\left(x_{0}\right)=0 \end{array}$$ (18)

The pixel-wise decomposition contains a non-linear dependence on the prediction point *x* beyond the Taylor series, as a close root point *x*_0_ needs to be found. Thus the whole pixel-wise decomposition is not a linear, but a locally linear algorithm, as the root point *x*_0_ depends on the prediction point *x*.

**Fig 3:**
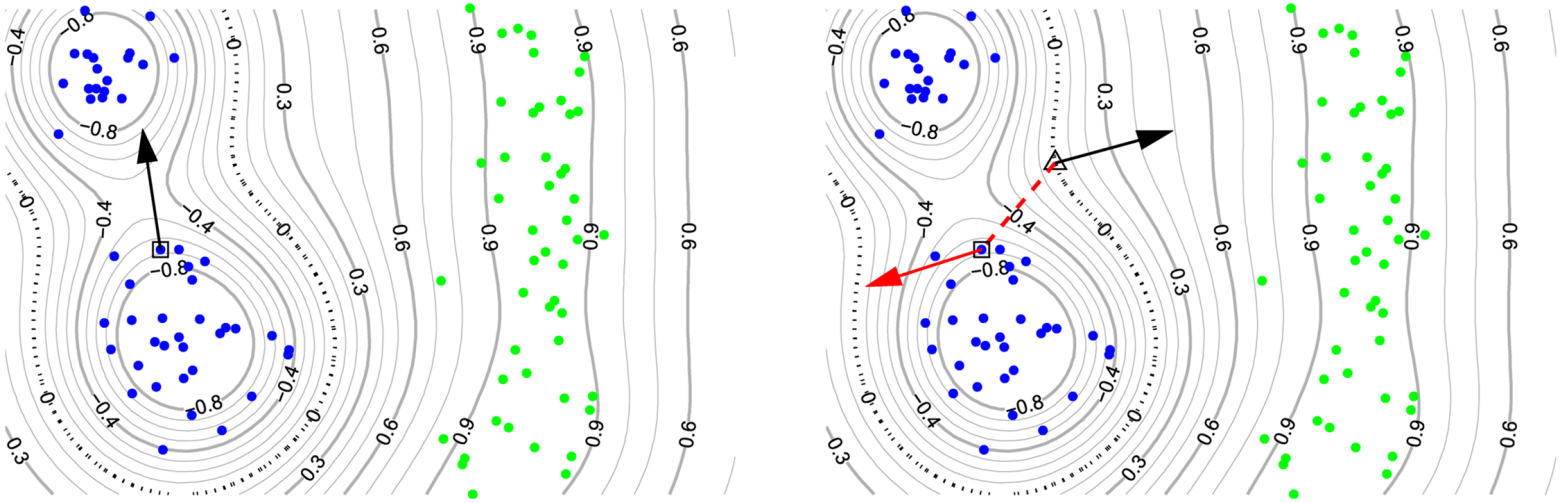
An exemplary real-valued prediction function for classification with the dashed black line being the decision boundary which separates the blue from the green dots. The blue dots are labeled negatively, the green dots are labeled positively. Left: Local gradient of the classification function at the prediction point. Right: Taylor approximation relative to a root point on the decision boundary. This figure depicts the intuition that a gradient at a prediction point *x*—here indicated by a square—does not necessarily point to a close point on the decision boundary. Instead it may point to a local optimum or to a far away point on the decision boundary. In this example the explanation vector from the local gradient at the prediction point *x* has a too large contribution in an irrelevant direction. The closest neighbors of the other class can be found at a very different angle. Thus, the local gradient at the prediction point *x* may not be a good explanation for the contributions of single dimensions to the function value *f*(*x*). Local gradients at the prediction point in the left image and the Taylor root point in the right image are indicated by black arrows. The nearest root point *x*_0_ is shown as a triangle on the decision boundary. The red arrow in the right image visualizes the approximation of *f*(*x*) by Taylor expansion around the nearest root point *x*_0_. The approximation is given as a vector representing the dimension-wise product between *Df*(*x*_0_) (the black arrow in the right panel) and *x* − *x*_0_ (the dashed red line in the right panel) which is equivalent to the diagonal of the outer product between *Df*(*x*_0_) and *x* − *x*_0_.

Several works have been using sensitivity maps [17–19] for visualization of classifier predictions which were based on using partial derivatives *at the prediction point *x**. There are two essential differences between sensitivity maps based on derivatives at the prediction point *x* and the pixel-wise decomposition approach. Firstly, there is no direct relationship between the function value *f*(*x*) at the prediction point *x* and the differential *Df*(*x*) at the same point *x*. Secondly, we are interested in explaining the classifier prediction relative to a certain state given by the set of roots of the prediction function *f*(*x*_0_) = 0. The differential *Df*(*x*) at the prediction point does not necessarily point to a root which is close under the Euclidean norm. It points to the nearest local optimum which may still have the same sign as the prediction *f*(*x*) and thus be misleading for explaining the difference to the set of root points of the prediction function. Therefore derivatives at the prediction point *x* are not useful for achieving our aim. Fig 3 illustrates the qualitative difference between local gradients (black arrows) and the dimension-wise decomposition of the prediction (red arrow).

One technical difficulty is to find a root point *x*_0_. For continuous classifiers we may use unlabeled test data in a sampling approach and perform a line search between the prediction point *x* and a set of candidate points {*x*′} such that their prediction has opposite sign: *f*(*x*)*f*(*x*′) < 0. It is clear that the line *l*(*a*) = *ax* + (1 − *a*)*x*′ must contain a root of *f* which can be found by interval intersection. Thus each candidate point *x*′ yields one root, and one may select a root point which minimizes the Taylor residuum or use an average over a subset of root points with low Taylor residues. A second possible solution would be, for example, to find the root point *x*_0_ that is the nearest to the data point *x*, or optimal in some measurable sense. However, while we have presented two types of solutions to finding a root point, in the most general case, the Taylor-type decomposition is best described as a constraint-based approach. In particular, the root point *x*_0_ at which the Taylor decomposition is computed is constrained to satisfy *f*(*x*_0_) = 0 and to lie not too far (e.g. within a fixed radius) from the actual data point *x*. Using this constraint-based definition, the most desirable properties of the Taylor decomposition are preserved, while the remaining specification is deferred to a later point in time.

Note that Taylor-type decomposition, when applied to one layer or a subset of layers, can be seen as an approximate way of relevance propagation when the function is highly non-linear. This holds in particular when it is applied to the output function *f* as a function of the preceding layer *f* = *f*(*z*_*i* − 1_), as Eq (18) satisfies approximately the propagation Eq (2) when the relevance of the output layer is initialized as the value of prediction function *f*(*x*). Unlike the Taylor approximation, layer-wise relevance propagation does not require to use a second point besides the input point. The formulas in Sections *Pixel-wise Decomposition for Classifiers over Bag of Words Features* and *Pixel-wise Decomposition for Multilayer Networks* will demonstrate that layer-wise relevance propagation can be implemented for a wide range of architectures without the need to approximate by means of Taylor expansion.

### Related Work

Several works have been dedicated to the topic of explaining neural networks, kernel-based classifiers in general and classifiers over Bag of Words features in particular.

As for neural networks, [20] is dedicated towards analyzing classifier decisions at neurons which is applicable also to the pixel level. It performs a layer-wise inversion down from output layers towards the input pixels for the architecture of convolutional networks [21]. This work is specific to the architecture of convolutional neural networks. Compared to our approach it is based on a different principle. See [22] which establishes an interpretation of the work in [20] as an approximation to partial derivatives with respect to pixels in the input image. In a high-level sense, the work in [20] uses the method from their own predecessor work in [23] which solves optimization problems in order to reconstruct the image input, while our approach attempts to reconstruct the classifier decision. In a more technical sense, the difference between [20] and our approach can be seen by comparing how the responses are projected down towards the inputs. [20] uses rectified linear units to project information from the unfolded maps towards the inputs with the aim to ensure the feature maps to be non-negative. In our approach we use the signed activations of the neurons from the layer below for weighting the relevance quantity from the layer above with the aim of conserving the relevance quantity across layers. The sign of the activations of neurons of the layer below, for which the relevance is to be computed, is used in our work for encoding assumptions about the structure of the neural network. With respect to neural networks, our work is applicable to a wide range of architectures. A different type of analysis for understanding neural networks deals with creating counter-intuitive examples, see for example the work in [24, 25]. For a treatment on how to discriminate structures which influence predictions from correlations with hidden variables such that the hidden variables have an impact on the predictor, see [26].

Another approach which lies between partial derivatives at the input point *x* and a full Taylor series around a different point *x*_0_ is presented in [22]. This work uses a different point *x*_0_ than the input point *x* for computing the derivative and a remainder bias which both are not specified further but avoids for an unspecified reason to use the full linear weighting term *x* − *x*_0_ of a Taylor series. Besides deviating in the usage of a Taylor series, the work in [22] does not make use of the layer-wise propagation strategy for neural networks presented in this paper, which unlike a Taylor series does not rely on a local approximation. Explanation of neural network behavior on the level of single neurons is done in [27] and [28]. These works try to find inputs which maximize the activation of neurons by means of optimization problems which can be solved by gradient ascent. Our approach aims at explaining the decision for a given input rather than finding optimal stimuli for a particular neuron. Note that the approach presented in our paper is different from plotting the activations of neurons during a forward-pass of the input image. The latter is independent from the neural network properties in higher layers whereas in our approach we feed the classification score into the top neurons and use quantities computed by using properties of higher layers to obtain a representation at lower layers. Quantifying the importance of input variables using a neural network model has also been studied in specific areas such as ecological modeling, where [29, 30] surveyed a large ensemble of possible analyses, including, computing partial derivatives, perturbation analysis, weights analysis, and studying the effect of including and removing variables at training time. A different avenue to understanding decisions in neural network is to fit a more interpretable model (e.g. decision tree) to the function learned by the neural network [31], and extract the rules learned by this new model.

With respect to sensitivity of kernel-based classifiers to input dimensions, [32] yields sensitivity maps, which are invariant to the sign of the predictions. [17–19] consider explanation vectors based on local gradients which are sign-sensitive. Both approaches use partial derivatives at the point which is to be predicted. The first works to our knowledge to visualize BoW models are [33] and [34]. The former work finds regions with a large influence for classification for the special case of max pooling, sparse coding and a linear classifier. The latter work obtains an exact decomposition into local feature scores for the case of a histogram intersection kernel and zero-one coding of local features onto visual words.

We differ from the above works on kernel-based classifiers over Bag of Words features in the following sense: Our methodology is applicable to *arbitrary* Bag of Words models including various feature codings such as Fisher vectors and regularized codings and to a broader class of kernels, namely all differentiable or so-called sum decomposable kernels. When relying on Taylor decomposition, our work relies on a Taylor series around a root close to the prediction point rather than partial derivatives at the prediction point itself. The rationale for this choice was justified in the preceding Section *Taylor-type decomposition*. Finally, in contrast to the preceding publications, our work introduces layer-wise relevance propagation for neural networks and Bag of Words features.

## Pixel-wise Decomposition for Classifiers over Bag of Words Features

Despite recent advances in neural networks, Bag of Words models are still popular for image classification tasks. They have excelled in past competitions on visual concept recognition and ranking such as Pascal VOC [35, 36] and ImageCLEF PhotoAnnotation [37]. In our experience Bag of Words Models perform well for tasks with small sample sizes, whereas neural networks are at risk to overfit due to their richer parameter structure.

### Bag of Words models revisited

We will consider here Bag of Words features as an aggregation of non-linear mappings of local features. All Bag of Words models, no matter whether based on hierarchical clustering [38], soft codebook mapping [15, 39–41], regularized local codings [12–14], or Fisher Vectors [11], share a multi-stage procedure in common.

In the first stage local features are computed across small regions in the image. A local feature such as SIFT [42, 43] is in the abstract sense a vector computed from a region of the image, for example capturing information of interest such as shape characteristics or properties of color or texture on a local scale. In a second stage which is performed once during training, representatives in the space of local features are computed, no matter whether they are cluster centroids obtained from k-means clustering, regions of the space as for clustering trees [38], or centers of distributions as for Fisher vectors [44]. The set of representatives, in the following referred to as *visual words*, serves as a vocabulary in the context of which images can be described as vectors. In the third stage, statistics of the local features are computed relative to those visual words. These statistics are aggregated from all local features *l* within an image in order to yield a BoW representation *x*, usually done by sum- or max-pooling.

The computation of statistics can be modeled by a mapping function accepting local feature vectors *l* as input, which are then projected into the Bag of Words feature space. Let *m* be such a mapping function and let *m*_(*d*)_ denote the mapping onto the *d*-th dimension of the BoW space. We assume the very generic p-means mapping scheme for local features *l* as given in Eq (19).

$$\begin{array}{c} x_{\left(d\right)}={\left(M^{-1}\sum_{j=1}^{M}{\left(m_{\left(d\right)}\left(l_{j}\right)\right)}^{p}\right)}^{\frac{1}{p}} \end{array}$$ (19)

This contains sum- and max-pooling as the special cases *p* = 1 and the limit *p* = ∞.

Finally, a classifier is applied on top of these features. Our method supports the general class of classifiers based on kernel methods. For brevity we use here an SVM prediction function which results in a prediction function over BoW features *x*_*i*_, training data labels *y*_*i*_, kernel functions *k*(⋅, ⋅), and SVM model parameters *b* and *α*_*i*_.

$$\begin{array}{c} f\left(x\right)=b+\sum_{i=1}^{S}\alpha_{i}y_{i}k\left(x_{i},x\right) \end{array}$$ (20)

This assumption can be extended without loss of generality to approaches using multiple kernel functions such as multiple kernel learning [45–49], structural prediction approaches with tensor product structure between features and labels as in taxonomy-based classifiers [50–52] or boosting-like formulations as in [53]

$$\begin{array}{c} f\left(x\right)=b+\sum_{i=1}^{S}\sum_{u=1}^{K}\alpha_{i,u}k_{u}\left(x_{i(u)},x_{\left(u\right)}\right). \end{array}$$ (21)

An overview over notations used for the Bag of Words features is given in Table 1.

**Table 1:** Notation Conventions Used in This Section.

*f*(⋅)	the classifier’s prediction function
*x*,*y*	BoW representation and class label
*x*_0_,*z*	root point of Taylor Expansion, root point candidates
*α*,*b*	learned model parameters
*k*(*x*_*i*_,*x*_*i*′_)	kernel function
*d*,*V*	counter and number of BoW-dimensions
$R_{d}^{\left(3\right)}$	(approximate) contribution of BoW-dimension *d*
$R_{l}^{\left(2\right)}$	local feature relevance
$R_{q}^{\left(1\right)}$	pixel-wise decompositions per pixel *q*
*m*(*l*)	mapping function between local features *l* and BoW
*l*,*l*′	local feature descriptors
*Z*(*x*)	the set of unmapped dimensions of a BoW data point *x*
area(*l*)	the set of pixel coordinates covered by *l*

### Overview of the decomposition steps

**Fig 4:**
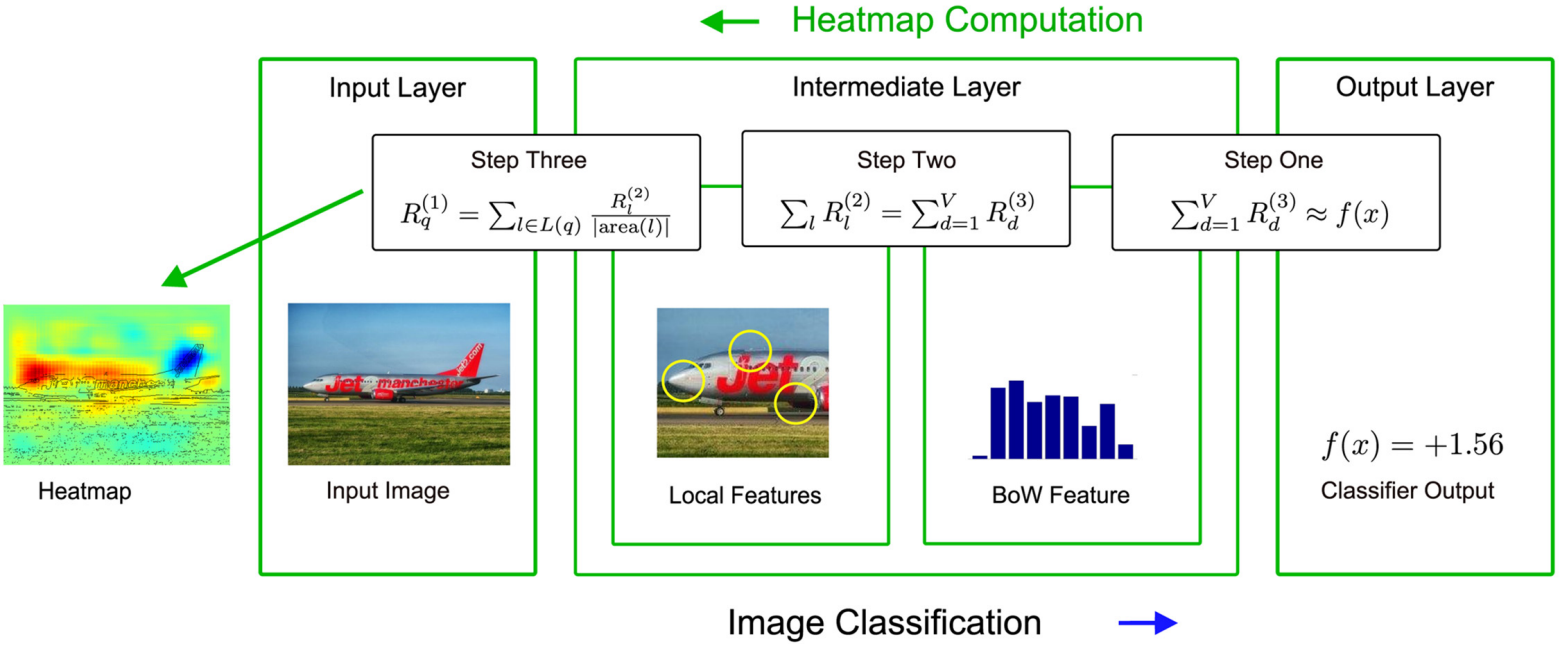
Local and global predictions for input images are obtained by following a series of steps through the classification- and pixel-wise decomposition pipelines. Each step taken towards the final pixel-wise decomposition has a complementing analogue within the Bag of Words classification pipeline. The calculations used during the pixel-wise decomposition process make use of information extracted by those corresponding analogues. Airplane image in the graphic by Pixabay user tpsdave.

The main contribution of this part is the formulation of a generic framework for retracing the origins of a decision made by the learned kernel-based classifier function for a BoW feature. This is achieved, in a broad sense as visualized in Fig 4, by following the construction of a BoW representation *x* of an image and the evaluation thereof by a classifier function in reverse direction. In this section we will derive a decomposition of a kernel-based classifier prediction into contributions of individual local features and finally single pixels. The proposed approach consists of three consecutive steps.

In the first step we will use, depending on the type of kernel, either the Taylor-type decomposition strategy or the layer-wise relevance propagation strategy. In the first step relevance scores $R_{d}^{\left(3\right)}$ for the third layer of the BoW feature extraction process are obtained, describing the influence of all BoW feature dimensions *d* by deconstructing the classifier prediction function *f*(*x*) such that $\sum_{d}R_{d}^{\left(3\right)}\approx f(x)$. In other words, we gain a decomposition into contributions $R_{d}^{\left(3\right)}$ describing *f*(*x*) as a sum of individual predictions for all dimensions of *x*.

In the second step we will apply the layer-wise relevance propagation strategy in order to obtain relevance scores $R_{l}^{\left(2\right)}$ for the local features *l* from the relevance scores $R_{d}^{\left(3\right)}$. Layer-wise relevance propagation ensures that $\sum_{l}R_{l}^{\left(2\right)}=\sum_{d}R_{d}^{\left(3\right)}\approx f(x)$ holds.

The third step describes the computation of pixel-wise scores $R_{q}^{\left(1\right)}$ from local feature relevance scores $R_{l}^{\left(2\right)}$, which are then visualized as heatmaps by color-coding.

#### Step one: relevance scores $R_{d}^{\left(3\right)}$ for the third layer of the BoW feature extraction process

The third layer is the BoW feature itself. In the first step we would like to achieve a decomposition of the classifier prediction *f*(*x*) into relevance scores $R_{d}^{\left(3\right)}$ for BoW feature dimension *d*.

$$\begin{array}{c} f\left(x\right)\approx \sum_{d=1}^{V}R_{d}^{\left(3\right)} \end{array}$$ (22)

The work of [34] has performed this step for the special case of one single histogram intersection kernel. Such a decomposition can be generalized naturally and performed without error for all kernel functions which are sum-decomposable along input dimensions. We define a kernel function *k* to be sum-decomposable if there exists kernel functions *k* ^(*d*)^ acting on single input feature dimensions such that

$$\begin{array}{c} k\left(x_{i},x_{i^{\prime }}\right)=\sum_{\left(d\right)}k^{\left(d\right)}\left(x_{i(d)},x_{i^{\prime }\left(d\right)}\right) \end{array}$$ (23)

In this case, as with the linear and histogram intersection kernel, we can achieve Eq (22) with equality in the following definition by applying layer-wise relevance propagation which results in Eq (24).

**Def. 1** ***Relevance scores for sum decomposable kernels***

$$\begin{array}{c} R_{d}^{\left(3\right)}=\frac{b}{V}+\sum_{i=1}^{S}\alpha_{i}y_{i}k^{\left(d\right)}\left(x_{i(d)},x_{\left(d\right)}\right) \end{array}$$ (24)

For the case of a general differentiable kernel we apply the Taylor-type decomposition strategy in order to linearly approximate the dimensional contributions $R_{d}^{\left(3\right)}$. The approximated dimensional contributions can be expressed as in Eq (25).

**Def. 2** ***Relevance scores for differentiable kernels***

$$\begin{array}{c} R_{d}^{\left(3\right)}:={\left(x-x_{0}\right)}_{\left(d\right)}\sum_{i=1}^{S}\alpha_{i}y_{i}\frac{\partial k(x_{i},\cdot )}{\partial x_{\left(d\right)}}\left(x_{0}\right) \end{array}$$ (25)

#### Step two: relevance scores $R_{l}^{\left(2\right)}$ for the second layer of the BoW feature extraction process

The second layer are the local features extracted from many regions of the image. In the second step we would like to achieve a decomposition of the classifier prediction *f*(*x*) into relevance scores $R_{l}^{\left(2\right)}$ for the local features *l* based on the relevances $R_{d}^{\left(3\right)}$ from the third layer.

For the sake of clarity, we do for now start with the case of sum-pooled BoW aggregation, to later extend to a more general formulation for p-means pooling from this point on.

As introduced in context of Eq (19) *m*_(*d*)_ denotes the mapping projecting to dimension *d* of the BoW space. We define the set of input dimensions *Z*(*x*) which are effectively not reached by the mappings of local features of an image. Applying the layer-wise propagation allows to define the local feature relevance score $R_{l}^{\left(2\right)}$ as given in Eq (27).

**Def. 3** ***Local feature scores for sum pooling***

$$\begin{array}{c} Z\left(x\right)=\{d|\sum_{l}m_{\left(d\right)}\left(l\right)=0\} \end{array}$$ (26)

$$\begin{array}{c} R_{l}^{\left(2\right)}:=\sum_{d\notin Z(x)}R_{d}^{\left(3\right)}\frac{m_{\left(d\right)}\left(l\right)}{\sum_{l^{\prime }}m_{\left(d\right)}\left(l^{\prime }\right)}+\sum_{d\in Z(x)}R_{d}^{\left(3\right)}\frac{1}{\left|\{l^{\prime }\}\right|} \end{array}$$ (27)

The coarse structure of definition (27) can be explained as taking the relevances from the layer above and weighting them with the outputs from the layer below. The summations over the mappings *m*_(*d*)_(*l*) over the local features {*l*} in Eq (27) achieve a weighting of the relevance from the third layer proportional to the ratios of the mappings. The second part describes an equal distribution of those relevance scores $R_{d}^{\left(3\right)}$ which correspond to dimensions of the Bag of Words feature *x* which have a value of zero and yet may contribute to the classifier prediction. To see this consider computing the *χ* ^2^-kernel value to a support vector which has a non-zero value in the same feature dimension. This is necessary to ensure that the propagation Eq (2) holds.

Summing the local feature relevance scores $R_{l}^{\left(2\right)}$ from Eq (27) yields the Taylor approximation $R_{d}^{\left(3\right)}$ of the prediction score *f*(*x*) in Eqs (24) or (25). This property is the key to our approach. We obtain exact summation to the prediction *f*(*x*) in the case of sum decomposable kernels and usage of Eq (24) in this special case.

$$\begin{array}{c} \sum_{l}R_{l}^{\left(2\right)}=\sum_{d=1}^{V}R_{d}^{\left(3\right)}\approx f\left(x\right) \end{array}$$ (28)

We would like to point out that this property holds also in the case when mappings *m*_(*d*)_ can become negative as a consequence of the definition used in (26) and (27), as can be seen from the summation given in the appendix. For that reason our approach is also applicable to Fisher vectors [11] and regularized coding approaches [12–14]. Furthermore note that definition (27) has no explicit dependence on the way how the local features are pooled in Eq (19) and this might be inappropriate weighting for max-pooling or general p-means pooling.

We can extend this definition to reflect the usage of p-means pooling

$$\begin{array}{cc} M_{p}\left(x_{1},\ldots ,x_{n}\right) & ={\left(\frac{1}{n}\sum_{i=1}^{n}x_{i}^{p}\right)}^{1/p} \end{array}$$ (29)

which may yield different results in prediction and local decomposition than sum pooling. The extension is well-defined for non-negative mappings *m*_(*d*)_ ≥ 0 and any value of *p* and for arbitrary mappings when combined with a value of *p* from the natural numbers.

**Def. 4** ***Local feature scores for p-means pooling***

$$\begin{array}{c} Z^{\left(p\right)}\left(x\right)=\{d|\sum_{l}m_{\left(d\right)}^{p}\left(l\right)=0\} \end{array}$$ (30)

$$\begin{array}{c} R_{l}^{\left(2\right)}:=\sum_{d\notin Z^{\left(p\right)}\left(x\right)}R_{d}^{\left(3\right)}\frac{m_{\left(d\right)}^{p}\left(l\right)}{\sum_{l^{\prime }}m_{\left(d\right)}^{p}\left(l^{\prime }\right)}+\sum_{d\in Z^{\left(p\right)}\left(x\right)}R_{d}^{\left(3\right)}\frac{1}{\left|\{l^{\prime }\}\right|} \end{array}$$ (31)

The first quotient in Eq (31) converges to an indicator function for the maximal mapping element in the limit *p* → ∞ which is consistent to max-pooling

$$\begin{array}{c} \frac{m_{\left(d\right)}^{p}\left(l\right)}{\sum_{l^{\prime }}m_{\left(d\right)}^{p}\left(l^{\prime }\right)}\to {𝕀}_{\left\{{\text{argmax}}_{l^{\prime }}m_{\left(d\right)}\left(l^{\prime }\right)\right\}}\left(l\right) \end{array}$$ (32)

#### Step three: relevance scores $R_{q}^{\left(1\right)}$ for the first layer of the BoW feature extraction process

The first layer are the pixels of the image. In order to calculate scores for each pixel we make use of information regarding local feature geometry and location known from the local feature extraction phase at the beginning of the image classification pipeline. The pixel score $R_{q}^{\left(1\right)}$ of an image coordinate *q* is calculated as a sum of local feature scores of all local features *l* covering *q*, weighted by the number of pixels covered by each local feature *l*. In terms of layer-wise relevance propagation a local feature is a computation unit which has as much inputs as the number of pixels it is covering. Without assumption of any further structure we distribute the relevance of the local feature equally to all its covered pixels. Layer-wise propagation yields Eq (34).

$$\begin{array}{c} L\left(q\right)=\{l\;|\;q\in \text{area}(l)\} \end{array}$$ (33)

$$\begin{array}{c} R_{q}^{\left(1\right)}=\sum_{l\in L(q)}\frac{R_{l}^{\left(2\right)}}{\left|\text{area}(l)\right|} \end{array}$$ (34)

The operator area(*l*) used Eqs (33) and (34) returns the set of pixel coordinates covered by a local feature *l*. Projecting the obtained scores $R_{q}^{\left(1\right)}$ to their respective image coordinates yields the pixel-wise decomposition representation *h* of the evaluated image.

For visualization in the sense of color coding, the pixel-wise decomposition *R* ^(1)^ is then normalized as

$$\begin{array}{c} R_{q}^{\prime (1)}=\frac{R_{q}^{\left(1\right)}}{\max_{q^{\prime }}\left(\right|R_{q^{\prime }}^{\left(1\right)}\left|\right)} \end{array}$$ (35)

ensuring that $\forall q:R_{q}^{\prime (1)}\in [-1,1]$. The normalized pixel-wise decomposition is then color coded by mapping the pixel scores to a color space of choice. In the case of individually classified sub-regions originating from the same image, a global pixel-wise decomposition can be constructed by averaging local pixel-wise decompositions scores.

Note that by choosing above normalization scheme, the assumption is made that at least one class is represented within the image. In case the assumption holds this might lead to prominent local predictions of even weakly projected features which we found suitable for the purpose of detecting class evidence. If this assumption does not hold, then images may display score artifacts dominated by the set of pixels covered by a small subset of local features which would otherwise be considered input noise. A solution for this problem is global normalization which uses a maximum over pixels over a set of images instead of one image. We found that a global normalization scheme can be more appropriate for visualizing the actual decision process of the classifier, as it preserves the relative order of magnitude of local feature scores in between pixel-wise decomposition tiles. Algorithm 1 gives an overview how to compute the pixel-wise decomposition for classifiers based on Bag of Words features and support vector machines.

**Algorithm 1** Pixel-wise decomposition for BoW features with SVM classifiers

**Inputs:**

Image *I*

Local features *L*

BoW representation *x* (and Taylor root point *x*_0_)

model and mapping parameters

**for** *d* = 1 **to** *V* **do**

$R_{d}^{\left(3\right)}$ as in Eqs (24) or (25)

**end for**

**for all** *l* ∈ *L* **do**

$R_{l}^{\left(2\right)}$ as in Eqs (27) or (31)

**end for**

**for all** pixels *q* ∈ *I* **do**

$R_{q}^{\left(1\right)}$ as in Eq (34)

**end for**

**Output:** $\forall q:\;R_{q}^{\left(1\right)}$

### Examples for various mappings and kernels

In order to illustrate the generality of this framework we give some examples for various methods of mapping local features and kernels.

#### Example case: Soft codebook mapping

A soft codebook mapping like in [15, 40] fits trivially into the framework

$$\begin{array}{c} m_{\left(d\right)}\left(l\right)=\frac{\exp(-\beta d\left(l,b_{d}\right))}{\sum_{\left(d\right)}\exp\left(-\beta d\left(l,b_{d}\right)\right)} \end{array}$$ (36)

#### Example case: Regularized coding

Considering formulations as in [12, 14]

$$\begin{array}{c} {\text{argmin}}_{B,C}\sum_{j}{∥l_{j}-Bc_{j}∥}^{2}+Q\left(c_{j}\right) \end{array}$$ (37)

where *B* is the codebook and *Q* denotes a regularizer on the codebook coefficients such as the ℓ_1_-norm *Q*(*c*) = ∑_*j*_∣*c*(*j*)∣, we arrive at

$$\begin{array}{c} m\left(l_{j}\right):=c_{j} \end{array}$$ (38)

where the *d*-th dimension of the so defined mapping *m*_(*d*)_ corresponds to the *d*-th basis element in *B*.

#### Example case: Fisher Vectors

The Fisher vector [44] can be replicated using the following mapping *m*_(*d*)_ given below. It is expected to be used with a linear kernel, thus we can use the exact formula from Eq (24) for defining the BoW dimension relevance scores $R_{d}^{\left(3\right)}$. We stick to the formulation of Fisher vectors as given in [11]. The difference to other mappings is that a BoW feature dimension corresponds to a derivative of a Gaussian mixture center with respect to some parameter and not to a visual word.

Let *g*_*k*_(*l*) be the *D*-dimensional Gaussian mixture component with diagonal covariance *σ*_*k*_ and mean *μ*_*k*_. We define the softmax of the GMM mixture parameters *α*_*k*_ as

$$\begin{array}{c} w_{k}=\frac{\exp(\alpha_{k})}{\sum_{j=1}^{K}\exp\left(\alpha_{j}\right)} \end{array}$$ (39)

Let the soft assignment of local feature *l* to Gaussian *k* be given as

$$\begin{array}{c} \gamma \left[l\right]\left(k\right)=\frac{w_{k}u_{k}\left(l\right)}{\sum_{j=1}^{K}w_{j}u_{j}\left(l\right)} \end{array}$$ (40)

Let *k* denote the index of a Gaussian mixture component, then the mapping contains three cases depending on whether we consider the derivative for the mixture parameter *α*_*k*_ stored in dimension *d* = (2*D* + 1)(*k* − 1) + 1, derivatives for the Gaussian mean parameter *μ*_*k*_ stored in dimensions *d* = (2*D* + 1)(*k* − 1) + 1 + *r*,*r* ∈ [1,*D*], or finally, derivatives for the Gaussian variance parameter *σ*_*k*_ stored in dimensions *d* = (2*D* + 1)(*k* − 1) + 1 + *D* + *r*,*r* ∈ [1,*D*]

We assume one kernel without loss of generality, *V*_1_ = (2*D* + 1)*K* where *K* is the number of Gaussian mixture components and *D* is the local feature dimension.

$$\begin{array}{c} m_{\left(d\right)}\left(l\right)=\{\begin{array}{cc} \frac{1}{\sqrt{w_{k}}}\left(\gamma \left[l\right]\left(k\right)-w_{k}\right) & \text{if}\;d=(2D+1)(k-1)+1\\ \frac{1}{\sqrt{w_{k}}}\gamma \left[l\right]\left(k\right)\frac{l_{r}-\mu_{k}}{\sigma_{k}} & \text{if}\;d=(2D+1)(k-1)+1+r,r\in [1,D]\\ \frac{1}{\sqrt{w_{k}}}\gamma \left[l\right]\left(k\right)\frac{1}{\sqrt{2}}\left(\frac{{\left(l_{r}-\mu_{k}\right)}^{2}}{\sigma_{k}^{2}}-1\right) & \text{if}\;d=(2D+1)(k-1)+1+D+r,r\in [1,D] \end{array} \end{array}$$ (41)

This definition can be extended in a straightforward manner to use power normalization and ℓ_2_ normalization from [11]. The difference to the soft mapping case from Section *Example case: Soft codebook mapping* is that a the *d*-th dimension of the mapping *m*_(*d*)_ corresponds not to a visual word but to a derivative with respect to a Gaussian mixture component.

#### Example case: Histogram intersection kernel

The histogram intersection kernel applies to the exact decomposition formula in Eq (24). It is defined as

$$\begin{array}{c} k_{\text{HI}}\left(x_{i},x_{i^{\prime }}\right)=\sum_{d}\min(x_{i(d)},x_{i^{\prime }\left(d\right)}) \end{array}$$ (42)

Plugging the kernel into (24) yields

$$\begin{array}{c} R_{d}^{\left(3\right)}=\frac{b}{V}+\sum_{i=1}^{S}\alpha_{i}y_{i}\min(x_{i(d)},x_{\left(d\right)}) \end{array}$$ (43)

#### Example case: *χ* ^2^ kernel

The *χ* ^2^ kernel function has been applied with good performance in various image classification tasks [41, 54, 55]. The *χ* ^2^ kernel is defined as

$$\begin{array}{c} k_{\chi^{2}}\left(x_{i},x_{i^{\prime }}\right)=\exp(-\sigma \sum_{d}\frac{{\left(x_{i(d)}-x_{i^{\prime }\left(d\right)}\right)}^{2}}{\left(x_{i(d)}+x_{i^{\prime }\left(d\right)}\right)}) \end{array}$$ (44)

To avoid divisions by zero in case of *x*_*i*(*d*)_ = *x*_*i*′(*d*)_ = 0 we adhere to the convention that $\frac{0}{0}=0$ for the kernel function itself, as well as its derivative

$$\begin{array}{c} \frac{\partial k_{\chi^{2}}\left(x_{i},x_{i^{\prime }}\right)}{\partial x_{i^{\prime }\left(d\right)}}=k_{\chi^{2}}\left(x_{i},x_{i^{\prime }}\right)\sigma (\frac{4{\left(x_{i(d)}\right)}^{2}}{{\left(x_{i(d)}+x_{i^{\prime }\left(d\right)}\right)}^{2}}-1) \end{array}$$ (45)

When plugged into Eq (25), the dimensional contributions of the *χ* ^2^ kernel are then obtained as

$$\begin{array}{c} R_{d}^{\left(3\right)}:={\left(x-x_{0}\right)}_{\left(d\right)}\cdot \sum_{i=1}^{S}\alpha_{i}y_{i}k_{\chi^{2}}\left(x_{i},x_{0}\right)\sigma \left(\frac{4{\left(x_{i(d)}\right)}^{2}}{{\left(x_{i(d)}+x_{0(d)}\right)}^{2}}-1\right) \end{array}$$ (46)

#### Example case: Gaussian-RBF kernel

The Gaussian-RBF kernel function is widely used in different communities and is one of the most prominent, if not the most prominent non-linear kernel function. The kernel function is defined as

$$\begin{array}{c} k_{\text{Gauss}}\left(x_{i},x_{i^{\prime }}\right)=\exp(-\frac{{∥x_{i}-x_{i^{\prime }}∥}_{2}^{2}}{\sigma }) \end{array}$$ (47)

Deriving the kernel function w.r.t *x*_*i*′(*d*)_ and plugging it into Eq (25) yields

$$\begin{array}{c} \frac{\partial k_{\text{Gauss}}\left(x_{i},x_{i^{\prime }}\right)}{\partial x_{i^{\prime }\left(d\right)}}=k_{\text{Gauss}}\left(x_{i},x_{i^{\prime }}\right)(\frac{2x_{i(d)}-2x_{i^{\prime }\left(d\right)}}{\sigma }) \end{array}$$ (48)

and consequently

$$\begin{array}{c} R_{d}^{\left(3\right)}:={\left(x-x_{0}\right)}_{\left(d\right)}\cdot \sum_{i=1}^{S}\alpha_{i}y_{i}k_{\text{Gauss}}\left(x_{i},x_{0}\right)(\frac{2x_{i(d)}-2x_{0(d)}}{\sigma }) \end{array}$$ (49)

## Pixel-wise Decomposition for Multilayer Networks

Multilayer networks are commonly built as a set of interconnected neurons organized in a layer-wise manner. They define a mathematical function when combined to each other, that maps the first layer neurons (input) to the last layer neurons (output). We denote each neuron by *x*_*i*_ where *i* is an index for the neuron. By convention, we associate different indices for each layer of the network. We denote by “∑_*i*_” the summation over all neurons of a given layer, and by “∑_*j*_” the summation over all neurons of another layer. We denote by *x*_(*d*)_ the neurons corresponding to the pixel activations (i.e. with which we would like to obtain a decomposition of the classification decision). A common mapping from one layer to the next one consists of a linear projection followed by a non-linear function:

$$\begin{array}{c} z_{ij}=x_{i}w_{ij}, \end{array}$$ (50)

$$\begin{array}{c} z_{j}=\sum_{i}z_{ij}+b_{j}, \end{array}$$ (51)

$$\begin{array}{c} x_{j}=g\left(z_{j}\right), \end{array}$$ (52)

where *w*_*ij*_ is a weight connecting the neuron *x*_*i*_ to neuron *x*_*j*_, *b*_*j*_ is a bias term, and *g* is a non-linear activation function. Multilayer networks stack several of these layers, each of them, composed of a large number of neurons. Common non-linear functions are the hyperbolic tangent *g*(*t*) = tanh(*t*) or the rectification function *g*(*t*) = max(0,*t*). This formulation of neural network is general enough to encompass a wide range of architectures such as the simple multilayer perceptron [56] or convolutional neural networks [57], where convolution and sum-pooling are linear operations.

### Taylor-type decomposition

Denoting by *f*:ℝ^*M*^ ↦ ℝ^*N*^ the vector-valued multivariate function implementing the mapping between input and output of the network, a first possible explanation of the classification decision *x* ↦ *f*(*x*) can be obtained by Taylor expansion at a near root point *x*_0_ of the decision function *f*:

$$\begin{array}{c} R_{d}^{\left(1\right)}={\left(x-x_{0}\right)}_{\left(d\right)}\cdot \frac{\partial f}{\partial x_{\left(d\right)}}\left(x_{0}\right) \end{array}$$ (53)

The derivative ∂*f*(*x*)/∂*x*_(*d*)_ required for pixel-wise decomposition can be computed efficiently by reusing the network topology using the backpropagation algorithm [56]. In particular, having backpropagated the derivatives up to a certain layer *j*, we can compute the derivative of the previous layer *i* using the chain rule:

$$\begin{array}{c} \frac{\partial f}{\partial x_{i}}=\sum_{j}\frac{\partial f}{\partial x_{j}}\cdot \frac{\partial x_{j}}{\partial x_{i}}=\sum_{j}\frac{\partial f}{\partial x_{j}}\cdot w_{ij}\cdot g^{\prime }\left(z_{j}\right). \end{array}$$ (54)

A requirement of the Taylor-based decomposition is to find roots *x*_0_ (i.e. points on the classification boundary) that support a local explanation of the classification decision for *x*. These roots can be found by local search in the neighborhood of *x*. However, as noted in [24], this can lead to points of the input space that are perceptually equivalent to the original sample *x* and whose choice as a root would produce non-informative pixel-wise decompositions.

Alternatively, root points can be found by line search on the segment defined by *x* and its closest neighbor of a different class. This solution is problematic when the data manifold is sparsely populated, as it is the case for natural images. In such case, it is likely that following a straight line between *x* and its nearest neighbor will strongly depart from the data manifold and produce roots *x*_0_ with similarly poor pixel-wise decompositions.

### Layer-wise relevance backpropagation

**Fig 5:**
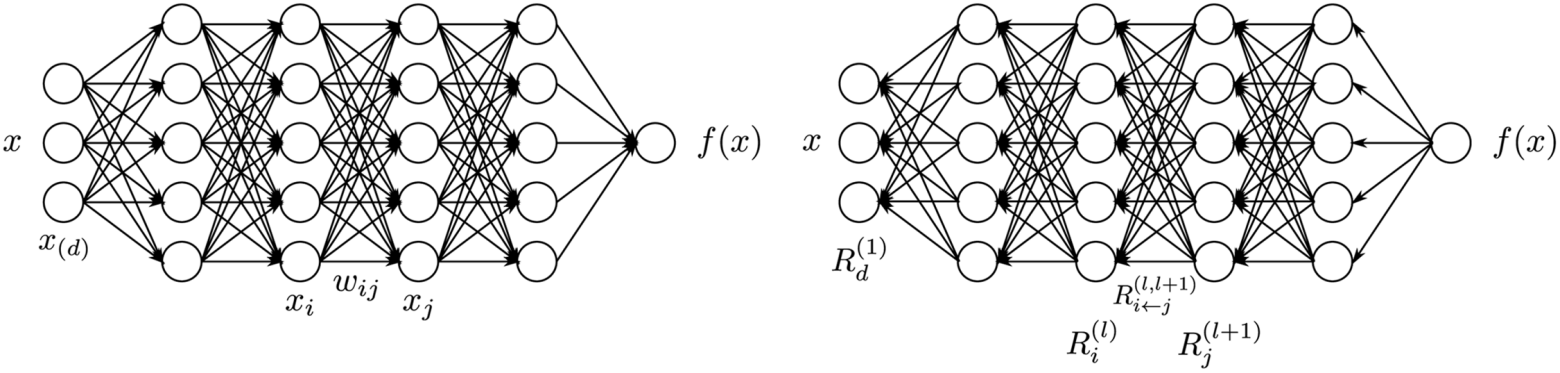
Multilayer neural network annotated with the different variables and indices describing neurons and weight connections. Left: forward pass. Right: backward pass.

As an alternative to Taylor-type decomposition, it is possible to compute relevances at each layer in a backward pass, that is, express relevances $R_{i}^{\left(l\right)}$ as a function of upper-layer relevances $R_{j}^{\left(l+1\right)}$, and backpropagating relevances until we reach the input (pixels). Fig 5 depicts a graphic example.

The method works as follows: Knowing the relevance of a certain neuron $R_{j}^{\left(l+1\right)}$ for the classification decision *f*(*x*), one would like to obtain a decomposition of such relevance in terms of messages sent to neurons of the previous layers. We call these messages *R*_*i* ← *j*_. In particular, as expressed by Eqs (8) and (13), the conservation property

$$\begin{array}{c} \sum_{i}R_{i\leftarrow j}^{\left(l,l+1\right)}=R_{j}^{\left(l+1\right)} \end{array}$$ (55)

must hold. In the case of a linear network *f*(*x*) = ∑_*i*_ *z*_*ij*_ where the relevance *R*_*j*_ = *f*(*x*), such decomposition is immediately given by *R*_*i* ← *j*_ = *z*_*ij*_. However, in the general case, the neuron activation *x*_*j*_ is a non-linear function of *z*_*j*_. Nevertheless, for the hyperbolic tangent and the rectifying function—two simple monotonically increasing functions satisfying *g*(0) = 0, —the pre-activations *z*_*ij*_ still provide a sensible way to measure the relative contribution of each neuron *x*_*i*_ to *R*_*j*_. A first possible choice of relevance decomposition is based on the ratio of local and global pre-activations and is given by:

$$\begin{array}{c} R_{i\leftarrow j}^{\left(l,l+1\right)}=\frac{z_{ij}}{z_{j}}\cdot R_{j}^{\left(l+1\right)} \end{array}$$ (56)

These relevances *R*_*i* ← *j*_ are easily shown to approximate the conservation properties of Eq (2), in particular:

$$\begin{array}{c} \sum_{i}R_{i\leftarrow j}^{\left(l,l+1\right)}=R_{j}^{\left(l+1\right)}\cdot (1-\frac{b_{j}}{z_{j}}) \end{array}$$ (57)

where the multiplier accounts for the relevance that is absorbed (or injected) by the bias term. If necessary, the residual bias relevance can be redistributed onto each neuron *x*_*i*_.

A drawback of the propagation rule of Eq (56) is that for small values *z*_*j*_, relevances *R*_*i* ← *j*_ can take unbounded values. Unboundedness can be overcome by introducing a predefined stabilizer *ɛ* ≥ 0:

$$\begin{array}{c} R_{i\leftarrow j}^{\left(l,l+1\right)}=\{\begin{array}{cc} \frac{z_{ij}}{z_{j}+\varepsilon }\cdot R_{j}^{\left(l+1\right)} & \;z_{j}\geq 0\\ \frac{z_{ij}}{z_{j}-\varepsilon }\cdot R_{j}^{\left(l+1\right)} & \;z_{j}<0 \end{array} \end{array}$$ (58)

The conservation law then becomes

$$\begin{array}{c} \sum_{i}R_{i\leftarrow j}^{\left(l,l+1\right)}=\{\begin{array}{cc} R_{j}^{\left(l+1\right)}\cdot (1-\frac{b_{j}+\varepsilon }{z_{j}+\varepsilon }) & \;z_{j}\geq 0\\ R_{j}^{\left(l+1\right)}\cdot (1-\frac{b_{j}-\varepsilon }{z_{j}-\varepsilon }) & \;z_{j}<0 \end{array} \end{array}$$ (59)

where we can observe that some further relevance is absorbed by the stabilizer. In particular, relevance is fully absorbed if the stabilizer *ɛ* becomes very large.

An alternative stabilizing method that does not leak relevance consists of treating negative and positive pre-activations separately. Let $z_{j}^{+}=\sum_{i}z_{ij}^{+}+b_{j}^{+}$ and $z_{j}^{-}=\sum_{i}z_{ij}^{-}+b_{j}^{-}$ where “ − ” and “+” denote the negative and positive part of *z*_*ij*_ and *b*_*j*_. Relevance propagation is now defined as

$$\begin{array}{c} R_{i\leftarrow j}^{\left(l,l+1\right)}=R_{j}^{\left(l+1\right)}\cdot (\alpha \cdot \frac{z_{ij}^{+}}{z_{j}^{+}}+\beta \cdot \frac{z_{ij}^{-}}{z_{j}^{-}}) \end{array}$$ (60)

where *α* + *β* = 1. For example, for *α*,*β* = 1/2, the conservation law becomes:

$$\begin{array}{c} \sum_{i}R_{i\leftarrow j}^{\left(l,l+1\right)}=R_{j}^{\left(l+1\right)}\cdot (1-\frac{b_{j}^{+}}{2z_{j}^{+}}-\frac{b_{j}^{-}}{2z_{j}^{-}}) \end{array}$$ (61)

which has similar form to Eq (57). This alternate propagation method also allows to control manually the importance of positive and negative evidence, by choosing different factors *α* and *β*.

Once a rule for relevance propagation has been selected, the overall relevance of each neuron in the lower layer is determined by summing up the relevance coming from all upper-layer neurons in consistence with Eqs (8) and (13):

$$\begin{array}{c} R_{i}^{\left(l\right)}=\sum_{j}R_{i\leftarrow j}^{\left(l,l+1\right)} \end{array}$$ (62)

The relevance is backpropagated from one layer to another until it reaches the input pixels *x*_(*d*)_, and where relevances $R_{d}^{\left(1\right)}$ provide the desired pixel-wise decomposition of the decision *f*(*x*). The complete layer-wise relevance propagation procedure for neural networks is summarized in Algorithm 2.

**Algorithm 2** Pixel-wise decomposition for neural networks

**Input:** *R* ^(*L*)^ = *f*(*x*)

**for** *l* ∈ {*L* − 1, …, 1} **do**

$R_{i\leftarrow j}^{\left(l,l+1\right)}$ as in Eqs (58) or (60)

$R_{i}^{\left(l\right)}=\sum_{j}R_{i\leftarrow j}^{\left(l,l+1\right)}$

**end for**

**Output:** $\forall d:\;R_{d}^{\left(1\right)}$

Above formulas (58) and (60) are directly applicable to layers which satisfy a certain structure. Suppose we have a neuron activation *x*_*j*_ from one layer which is modeled as a function of inputs from activations *x*_*i*_ from the preceding layer. Then layer-wise relevance propagation is directly applicable if there exists a function *g*_*j*_ and functions *h*_*ij*_ such that

$$\begin{array}{c} x_{j}=g_{j}(\sum_{i}h_{ij}\left(x_{i}\right)) \end{array}$$ (63)

In such a general case, the weighting terms *z*_*ij*_ = *x*_*i*_ *w*_*ij*_ from Eq (50) have to be replaced accordingly by a function of *h*_*ij*_(*x*_*i*_). A key observation is that relevance propagation is invariant against the choice of function *g*_*j*_ for computing relevances for the inputs *x*_*i*_ *conditioned on keeping the value of relevance *R*_*j*_ for *x*_*j*_ fixed*. This observation allows to deal with non-linear activation functions *g*_*j*_. The function *g*_*j*_ does however exert influence on computing the relevance *R*_*i*_ by its influence on the relevance *R*_*j*_ for *x*_*j*_. This can be explained by the fact, that the choice of *g*_*j*_ determines the value of *x*_*j*_ and thus also relevance *R*_*j*_ for *x*_*j*_ which gets assigned by the weights in the layer above.

We remark again, that even max pooling fits into this structure as a limit of generalized means, see Eq (32) for example. For structures with a higher degree of non-linearity, such as local renormalization [58, 59], Taylor approximation applied to neuron activation *x*_*j*_ can be used again to achieve an approximation for the structure as given in Eq (63).

Finally, it can be seen from the formulas established in this section that layer-wise relevance propagation is different from a Taylor series or partial derivatives. Unlike Taylor series, it does not require a second point other than the input image. Layer-wise application of the Taylor series can be interpreted as a generic way to achieve an approximate version of layer-wise relevance propagation. Similarly, in contrast to any methods relying on derivatives, differentiability or smoothness properties of neuron activations are not a necessary requirement for being able to define formulas which satisfy layer-wise relevance propagation. In that sense it is a more general principle.

## Experiments

For Bag of Words features we show two experiments, one on an artificial but easily interpretable data set and one for on Pascal VOC images which have a high compositional complexity. For the artificial data set we apply the Taylor-type strategy for the top layer, for Pascal VOC images we apply the strategy for sum-decomposable kernels for the top layer. In both cases these strategies are combined with our definitions for arbitrary mappings for the lower layers.

For neural networks we show results also on two data sets, two sets of results on MNIST which are easy to interpret, and a second set of experiments in which we rely on a 15 layers already trained network provided as part of the Caffe open source package [60], which predicts the 1000 categories from the ILSVRC challenge. On one side, by the experiments on MNIST digits, we intend to show that we can uncover details specific to the training phase. On the other side, the results for the pre-trained network from the Caffe toolbox demonstrate, that the method works with a deep neural network out of the box and does not rely on possible tricks during the training phase.

### Bag of Words features for polygons versus circles

**Fig 6:**
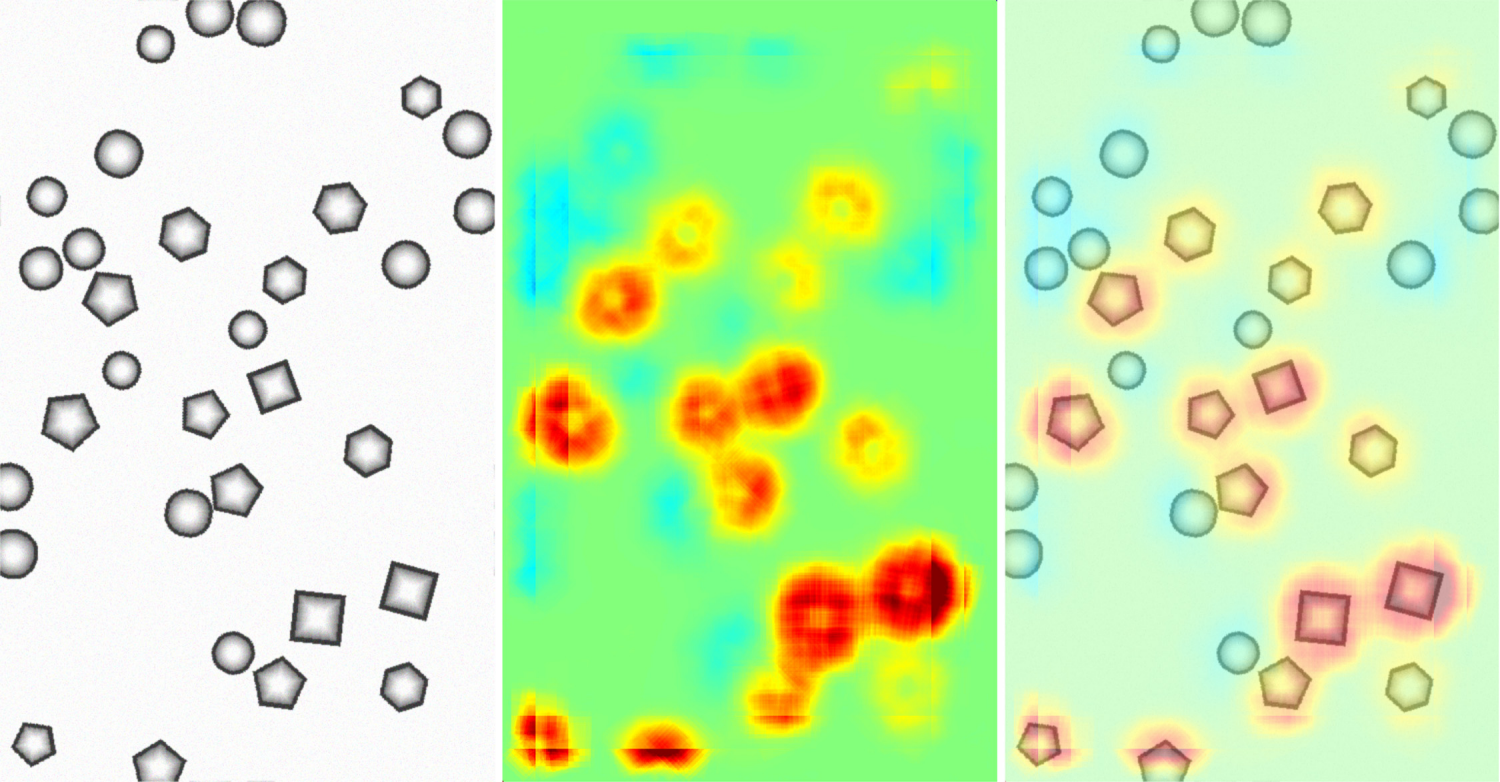
Pixel-wise decomposition for Bag of Words features over *χ* ^2^-kernels using the Taylor-type decomposition for the third layer and the layer-wise relevance propagation for the subsequent layers. Left: The original image. Middle: Pixel-wise prediction. Right: Superposition of the original image and the pixel-wise prediction. The decompositions were computed on tiles of size 102 × 102 and having a regular offset of 34 pixels. The decompositions from the overlapping tiles were averaged. In the heatmap, based on linearly mapping the interval [−1, +1] to the jet color map available in many visualization packages, green corresponds to scores close to zero, yellow and red to positive scores and blue color to negative scores. See text for interpretation.

An example of a pixel-wise decomposition for synthetic data is given in Fig 6. The training images were image tiles of size 102 × 102 pixels. An image was labeled positive if it contained at least one polygon independent of the presence of circles, and labeled negative if it contained no shape or circles only. The BoW features have been computed over standard SIFT features on gray scale brightness values on a dense grid with scales 2.0 and 3.0 resulting in one BoW feature for each scale. The SIFT feature masks have been rotated relatively to the main pixel gradient direction at their respective anchor locations. One *χ* ^2^-kernel as in Eq (44) was used for each BoW feature in conjunction with multiple kernel learning. Therefore the Taylor-type decomposition was used for computing the third layer relevances $R_{d}^{\left(3\right)}$ by Eq (46). The 128-dimensional BoW features are computed using a sum-pooled rank-mapping paradigm

$$\begin{array}{c} x_{\left(d\right)}=M^{-1}\sum_{j=1}^{M}m_{\left(d\right)}\left(l_{j}\right) \end{array}$$

$$\begin{array}{c} m_{\left(d\right)}\left(l\right)=\{\begin{array}{cc} p^{-{\text{rk}}_{d}\left(l\right)} & ;{\text{rk}}_{d}\left(l\right)\leq n\\ 0 & ;\text{else} \end{array} \end{array}$$ (64)

followed by an ℓ_1_-normalization of *x*. Similar as in [41] we set *p* = 2 and *n* = 4. The expression rk_*d*_(*l*) in Formula (64) describes the rank of the BoW prototype representing dimension *d* in the BoW feature space among ascendingly ordered Euclidean distances between *l* and all BoW prototypes. Thus Formula (27) based on layer-wise relevance propagation was used to obtain predictions $R_{l}^{\left(2\right)}$. In order to compute the visualization in Fig 6, the test picture was divided into tiles of sizes 102 × 102 with a grid step of 34 pixels. For each image tile one pixel-wise decomposition was computed. The overlapping pixel-wise decompositions were averaged, the resulting heatmap was normalized to the range [−1, +1] by dividing it by the maximal absolute pixel value.

The averaged classifier decomposition in Fig 6 shows firstly that the pixel-wise decomposition achieved by combining both decomposition strategies yields plausible results. The classifier making predictions on tiles was trained to discriminate polygons versus circles. The heatmap visualization of the pixel-wise decomposition is consistent with that result: Pixels close to polygons achieve positive pixel-wise scores, visible by the yellow to red colors. Pixels close to circles are mapped in light blue colors corresponding to negative pixel-wise scores. Pixels far away from shapes are in green which encodes scores close to zero.

This observation implies that local features over polygons are provided on average with positive second layer scores $R_{l}^{\left(2\right)}\gg 0$ and local features over circles receive on average weakly negative second layer scores $R_{l}^{\left(2\right)}<0$. Furthermore regions which are far from shapes result in scores close to zero which are colored in green in the heatmap from pixel-wise decomposition of the predictions and in the overlay of the image and the decomposition. We remark that the shapes have arbitrary positions in the image tiles of the training set and that position information was never used during training or BoW feature extractions. Thus, the pixel-wise decomposition is able to detect structures on a smaller scale than the classifier was trained on although position information was never provided during training or used in the classification algorithm which maps an image tile to a real-valued classifier prediction. The resolution of the smaller scale is limited by the scale of the SIFT features used here, the scale 3.0 results in a SIFT feature with side length of 4 ⋅ 3 ⋅ 3 = 36 pixels. This explains also why pixel scores outside of a shape but nearby to a shape receive the color which corresponds to the label induced by the neighboring shape.

Finally we remark that the rank-mapping is a discontinuous weighting scheme for BoW feature dimensions, yet the layer-wise propagation yields reasonable explanations.

### Bag of Words features for the Pascal VOC2009 data set

**Fig 7:**
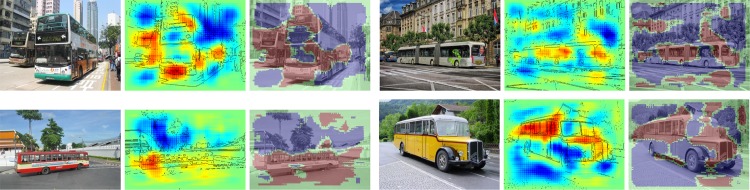
Pixel-wise decomposition for Bag of Words features over a histogram intersection kernel using the layer-wise relevance propagation for all subsequent layers and rank-mapping for mapping local features. Each triplet of images shows—from left to right—the original image, the pixel-wise predictions superimposed with prominent edges from the input image and the original image superimposed with binarized pixel-wise predictions. The decompositions were computed on the whole image. Images twice by Pixabay users tpsdave, and by Pixabay users sirocumo and Pixeleye.

**Fig 8:**
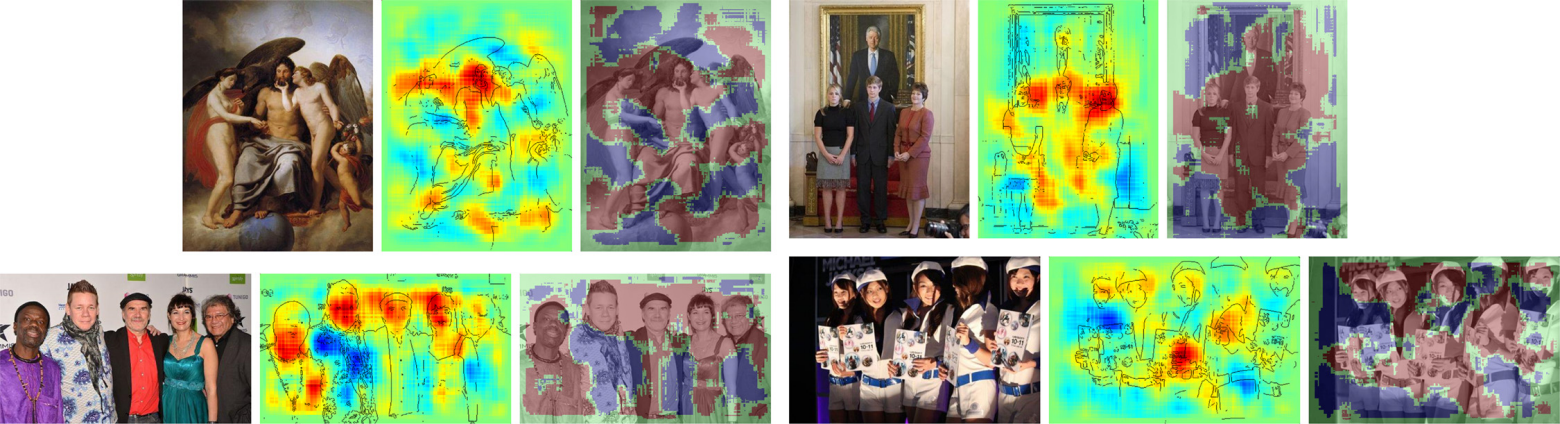
Pixel-wise decomposition for Bag of Words features over a histogram intersection kernel using the layer-wise relevance propagation for all subsequent layers and rank-mapping for mapping local features. Each triplet of images shows—from left to right—the original image, the pixel-wise predictions superimposed with prominent edges from the input image and the original image superimposed with binarized pixel-wise predictions. The decompositions were computed on the whole image. Faces below the hairline but also hands yield high scores, see the woman in the third picture which turns away her face from the camera as an example that hair alone is not relevant. Images by Pelagio Palagi, Wikimedia users Rorschach, Frankie Fouganthin and Flickr user Le vent dans les dunes.

**Fig 9:**
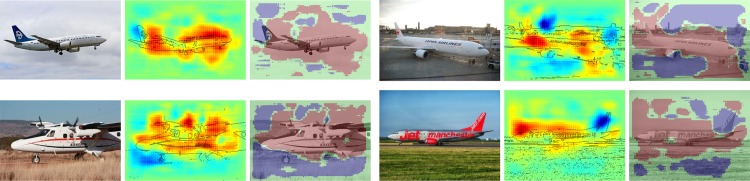
Pixel-wise decomposition for Bag of Words features over a histogram intersection kernel using the layer-wise relevance propagation for all subsequent layers and rank-mapping for mapping local features. Each triplet of images shows—from left to right—the original image, the pixel-wise predictions superimposed with prominent edges from the input image and the original image superimposed with binarized pixel-wise predictions. The decompositions were computed on the whole image. Notably the tail of a plane receives negative scores consistently. Blue sky context seems to contribute to classification which has been conjectured already in the PASCAL VOC workshops [35] and which was observed also on other images not shown here, see the the second picture for comparison against the other three images which have more blueish sky. Images from Pixabay users Holgi, nguyentuanhung, rhodes8043 and tpsdave.

**Fig 10:**
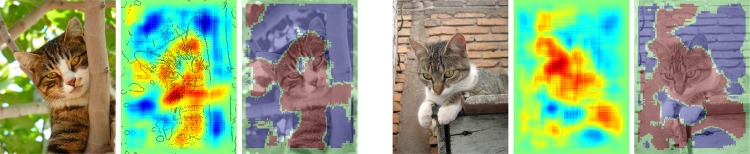
Pixel-wise decomposition for Bag of Words features over a histogram intersection kernel using the layer-wise relevance propagation for all subsequent layers and rank-mapping for mapping local features. Each triplet of images shows—from left to right—the original image, the pixel-wise predictions superimposed with prominent edges from the input image and the original image superimposed with binarized pixel-wise predictions. The decompositions were computed on the whole image. Positive responses seem to exist for certain fur texture patterns, see also the false responses on the wood and the plaster in the second example which both have similar texture and color to a cat’s fur. Images by Pixabay users LoggaWiggler and Holcan.

We have calculated pixel-wise predictions for images from the evaluation set of the Pascal VOC 2009 image classification challenge. The BoW representations of the training and test part of the data set have been computed over whole images based on local features extracted from a dense regular grid and fixed rotation. Standard SIFT features and stacks of 9-dimensional quantile features measuring values from 0.1 to 0.9 of the data over color intensities as in [41] have been used at local feature scale 3.0. The quantile features are split into two regions describing halves of a circle, yielding one 9-dimensional quantile over color intensities each, which are then concatenated. The SIFT feature descriptors have been computed once over a gray scale version of the image, stacked with feature descriptors computed over a combination if opposing color channels $\frac{\text{red}+\text{green}-2\cdot \text{blue}+2}{4}$, resulting in a 128+128 = 256 dimensional local feature vector. For SIFT as well as the color intensity quantiles, the same procedure has been applied to the red, green and blue color channels of the images separately, resulting in 128⋅3 = 384 and (2⋅9)⋅3 = 54 dimensions for the local features respectively. SIFT and quantile features were mapped onto 1024 dimensional and 512 dimensional BoW spaces respectively, using the ranked mapping scheme in Formula (64) with *p* = 2.4 and *n* = 8. Classifiers were trained for detecting the presence of the target object category regardless of the presence of other classes, using multiple kernel learning on three histogram intersection kernels, one for each computed local feature configuration. Consequently, the layer-wise relevance propagation Formulas (24) and (27) were used to compute local predictions for each evaluation image as a whole. Pixel-wise predictions for the classes representing buses, persons, airplanes and cats are presented in Figs 7, 8, 9 and 10 respectively. An interpretation which has been drawn from the depicted examples and other examples which are omitted here is given in the figure captions.

### Neural Networks for MNIST digits

We would like to investigate the capacity of relevance propagation to find evidence for classification of MNIST handwritten digits. A particular advantage of this data set over many-class image data sets is that it is easy for humans to interpret both positive and negative evidence, because of the small number of classes. For example, evidence for the handwritten digit “1” comes from the presence of a vertical bar on the pixel grid, but also from the absence of horizontal bar starting from the top of the vertical bar, which would make it a “7”. Also, the data set is relatively simple and the relation between the training algorithm and the resulting pixel-wise relevances can be analyzed.

We perform three experiments for MNIST data, as we would like to demonstrate that the method is able to uncover properties specific to the way of training. One experiment is done with a smaller network which is trained without translations of digits for the sake of allowing a direct comparison of the pixel-wise decomposition results to class-densities for each pixel of digits and seeing the impact of artifacts in the training set. Two further experiments are done on a larger network with has been trained without artifacts and with translated versions of digits and more training iterations for the sake of a better response to digits. The latter two experiments intend to show the impact of non-digit pixels as positive and negative evidence for a class of digits.

#### MNIST experiments *I*

The first set of experiments is done on a fully-connected neural network trained in the most common way: Input data is normalized so that the sum of pixels is on average zero, and the variance of pixel values is on average one. This setting implies that only black pixels yield strong inputs whereas white pixels fire only due to mean subtraction. The absence of translation invariance during training allows to uncover correlations of the pixel-wise decomposition to pixel-wise training densities and, as we will see in the experiments, allow to uncover artifacts in the data that may harm generalization.

**Fig 11:**
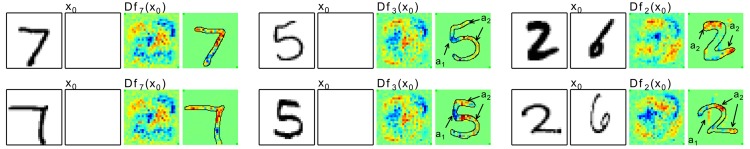
Taylor-approximated pixel-wise predictions for a multilayer neural network trained and tested on the MNIST data set. Each group of four horizontally aligned panels shows—from left to right—the input digit, the Taylor root point *x*_0_, the gradient of the prediction function *f* at *x*_0_ of a specific digit class indicated by the subscript next to *f* and the approximated pixel-wise contributions for *x*.

**Fig 12:**
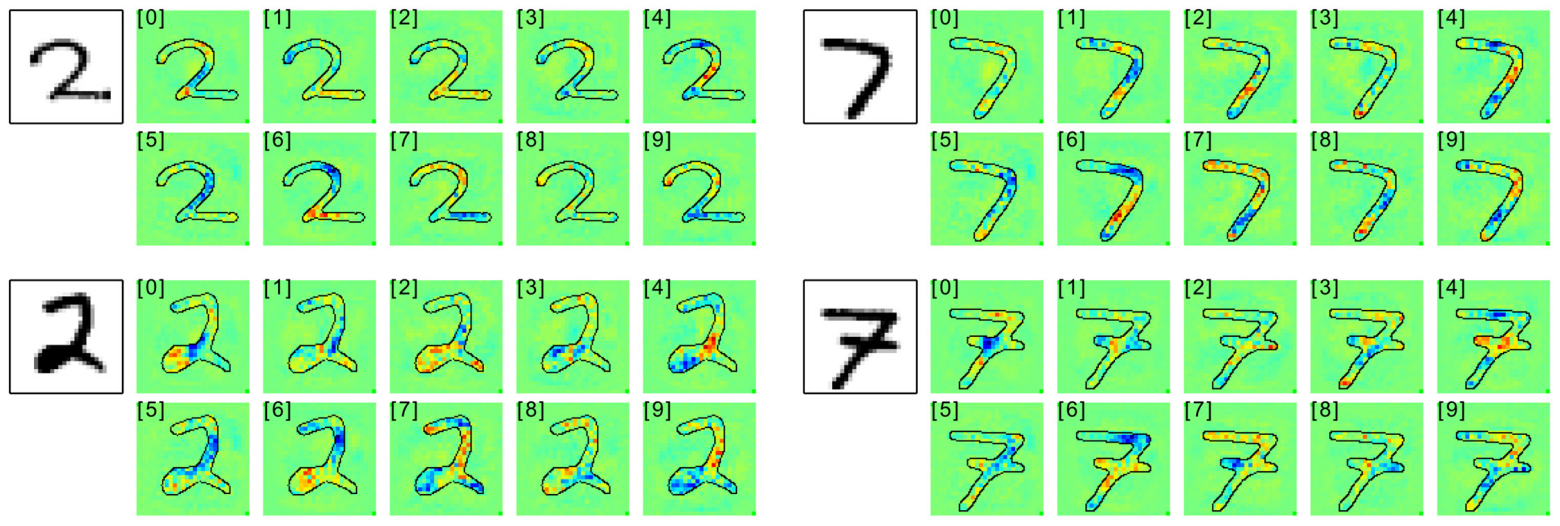
Pixel-wise decompositions for a multilayer neural network trained and tested on MNIST digits, using layer-wise relevance propagation as in Formula (56). Each group shows the decomposition of the prediction for the classifier of a specific digit indicated in parentheses.

**Fig 13:**
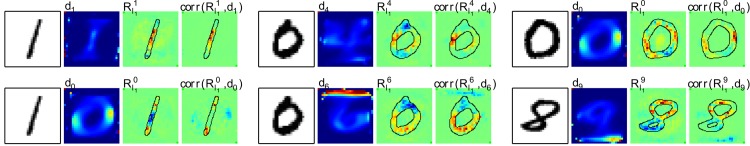
Each quadruple shows: on the leftmost the input digit; on the middle left the class specific pixel-wise density ratios *d*_*k*_ (Eq (65)) for the digit class *k* for which the pixel-wise decomposition is computed; on the middle right the pixel-wise decomposition *R* ^(1)^ for that digit and the digit class *k*; on the rightmost the correlation between *d*_*k*_ and the pixel-wise decomposition *R* ^(1)^. When considering a digit from class *i* and a pixel-wise decomposition from class *k* ≠ *i*, it is observable that the pixel-wise decomposition shows frequently highly positive activations on pixels of the digit from class *i* which have high relative density *d*_*k*_ for the digit class *k* ≠ *i*.

Examples for pixel-wise decompositions for the first type of neural networks are given in Figs 11, 12 and 13. Multilayer neural networks were trained on the MNIST [57] data of handwritten digits and solve the posed ten-class problem with a prediction accuracy of 98.25% on the MNIST test set. Our network consists of three linear sum-pooling layers with a bias-inputs, followed by an activation or normalization step each. The first linear layer accepts the 28 × 28 pixel large images as a 784 dimensional input vector and produces a 400-dimensional tanh-activated output vector. The second layer projects those 400 inputs to equally many tanh-activated outputs. The last layer then transforms the 400-dimensional space to a 10-dimensional output space followed by a softmax layer for activation in order to produce output probabilities for each class. The network was trained using a standard error back-propagation algorithm using batches of 25 randomly drawn training samples with an added Gaussian noise layer per training iteration. The above prediction accuracy was achieved after terminating the training procedure after 50 000 iterations.

Fig 11 shows pixel-wise relevances resulting from Taylor-type decomposition as defined in Eqs (18) and (53). The left column of the figure shows approximated pixel-wise relevances of the input digits, both times a 7, relative to a blank pixel tile. Relevances are calculated for the classifier output producing the prediction score for the class of digits 7. Evidently the upper part of the digit and most prominently the horizontal bar at the top are important characteristics for the predictor speaking for the digit’s class. The middle column of Fig 11 shows pixel-wise relevances for inputs from digit class 5 relative to a blank tile as interpreted by the classifier output reserved for the class of digit 3. As for digit class 7, the gradient at the root point *x*_0_ shows high positive prediction weights in areas corresponding to typical characteristics of the target class. We observe strong positive responses in areas shared by both digit classes 5 and 3 (indicated by annotation a_2_) and negative pixel-wise responses where the upper left vertical bar of the input digits is located, which is usually not present in a digit 3 (a_1_). The rightmost column of Fig 11 indicates distinguishing parts of the input digits from class 2 relative to their counter parts from digit class 6 chosen by minimal euclidean distance. High pixel scores highlight characteristics of the prediction point indicating membership in class 2, most notable the upper arc and the tail of the input digits 2, as marked with the annotation a_2_. Notably in the lower right image of the figure the absence of the leftmost vertical arc of the digit 6 (annotation a_1_) in the prediction point causes positive local predictions for class 2.

Pixel-wise predictions obtained via the layer-wise relevance propagation Formula (56) were calculated based on the output of the last linear layer without taking the succeeding softmax normalization layer into account. We can see pixel-wise decompositions for exemplary digits in Fig 12. Notably, for the pixel-wise decomposition of the digit 2, we see highly positive responses in lower parts of the digits for the classifiers for digits 2 and 6, however for the classifier for digit 6, the prediction score is negative, because the high positive responses in the lower part are suppressed by high negative responses in the upper part where the digit 2 has an arc which is not present in the digit 6.

Correlating the pixel-wise decompositions for classifier for digit *k* with the relative density of the pixels of the digits *k* in the training set demonstrates the plausibility of the decomposition.

$$\begin{array}{c} d_{k}\left(p\right)=\frac{\sum_{N\in \text{digits}(k)}N\left(p\right)}{\sum_{l=0}^{9}\sum_{N\in \text{digits}(l)}N\left(p\right)} \end{array}$$ (65)

This can be seen in Fig 13. When considering the decomposition for a classifier for digit *k* and using a digit *i* ≠ *k* from the wrong class as input, then we observe often that the decomposition shows high positive activations on those pixels of digit *i* which have high relative density *d*_*k*_(*p*) (Eq (65)) within the training set for digits of type *k*. Even when we use the classifier for the wrong class, it uncovers evidence on those pixels which overlap regions with high density of positively labeled digits of its training set.

#### MNIST experiments *I* *I*

In this set of experiments, we train larger neural networks on the MNIST data set augmented translated copies of the digits. Neural networks are composed of three hidden layers of 1296 units each, where weight connections between layers are initialized at random. Neural networks are trained by backpropagation using stochastic gradient descent with mini batches of size 25 and using a softmax objective [6]. In order to force the neural network to make decisions based on the shape of digits rather than their absolute position on the pixel grid, we augment the training data with pixel-wise translations of ±4 pixels (both vertical and horizontal) and add 10% salt-and-pepper noise to the inputs. Also, in order to favor the construction of evidence on both black and white pixels, MNIST digits are re-scaled to take pixel values between −1 (white pixel) and 1 (black pixel).

**Fig 14:**
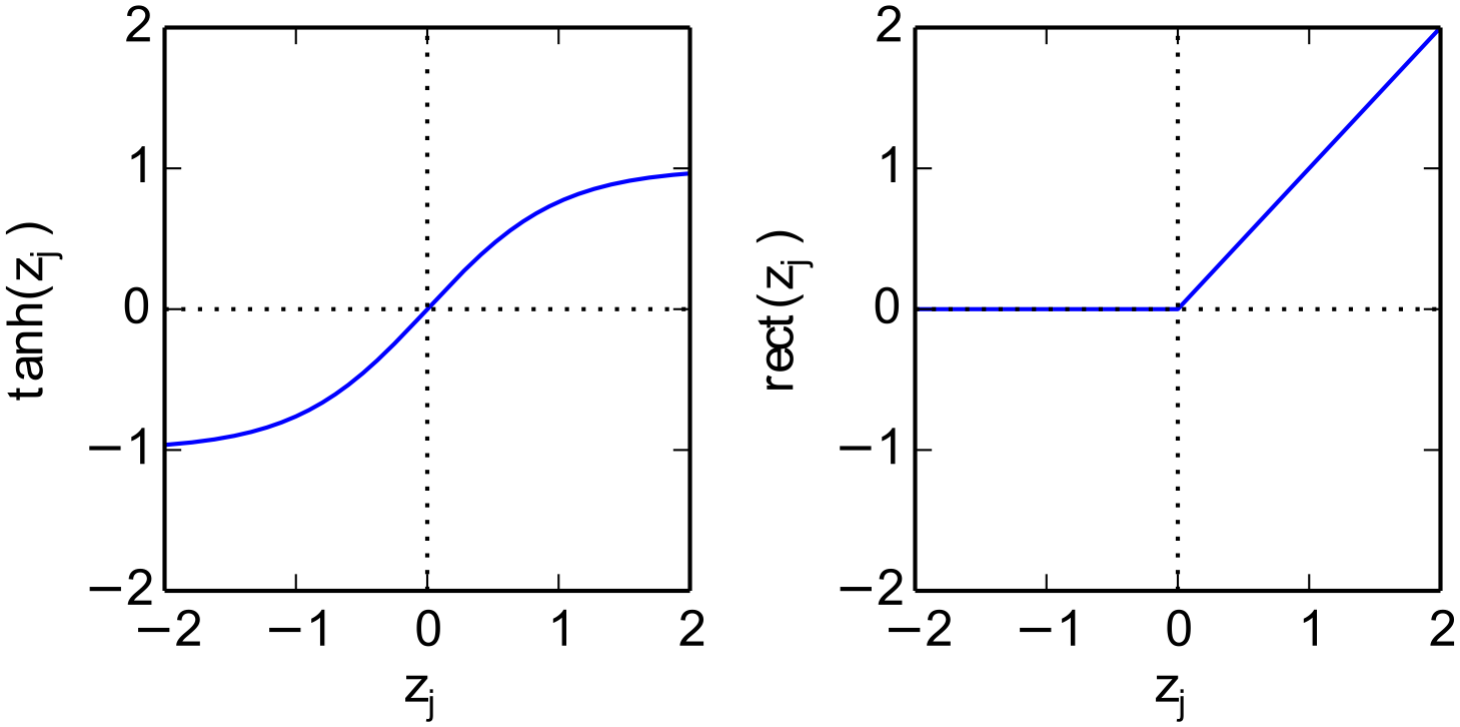
Example of non-linear activation functions *g* used in multilayer neural networks.

We consider two types of non-linearities: (1) rectified linear units and (2) hyperbolic tangent sigmoids. These non-linearities are some of the most commonly used in neural networks and are plotted in Fig 14. Also, in order to study explanations on different level of detail, we consider (1) a network that has been trained for 1 000 000 iterations until it reaches 99.2% accuracy and (2) a network trained for only 10 000 iterations, at which point it reaches approximately 97.0% accuracy.

**Fig 15:**
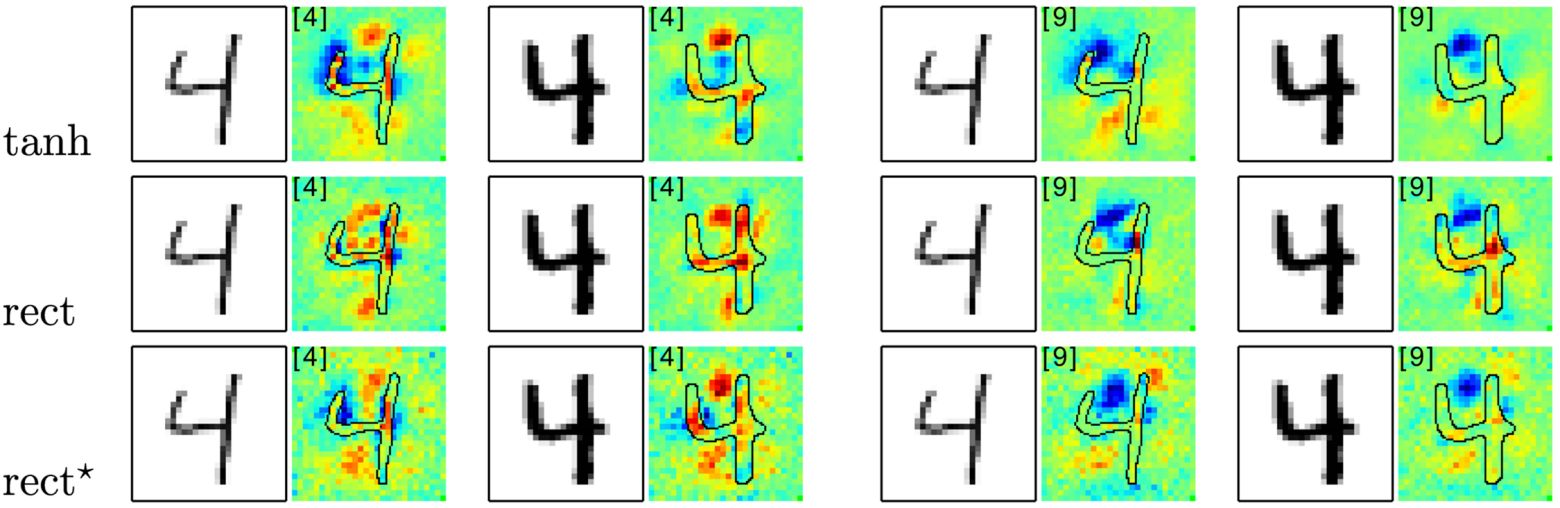
Evidence for a handwritten digit being a “4” or a “9”. Strong positive evidence for “4” is allocated to the top part of the image for keeping it blank. If trying to interpret these digits as “9”, the open top-part of the image is perceived as negative evidence for this class, because a “9” would rather have a top-dash closing the upper loop of the “4”. Explanations are consistent across a variety of neural networks and samples.

**Fig 16:**
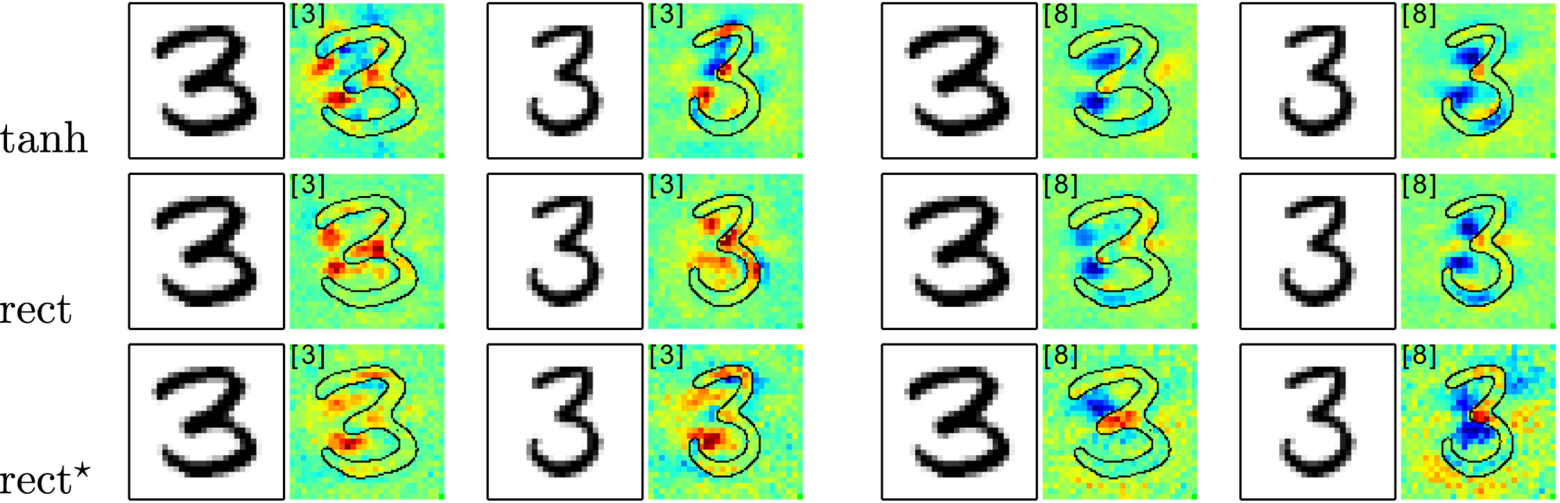
Evidence for a handwritten digit being a “3” or a “8”. Classifying as “3” is supported by the middle horizontal stroke featured in this digit and the absence of vertical connections on the left of the image. Evidence for being a “8” feature again the middle horizontal stroke, however, the absence of connections on the left side of the digit constitutes negative evidence. Explanations are again stable for various models and samples.

Figs 15 and 16 are two case studies of explanations produced by the neural networks we have trained. The number in brackets on the top-left corner of the pixel-wise decomposition indicates the class with respect to which evidence is measured. Similar explanations are produced by a network trained with rectified linear units. It is interesting to note that layer-wise relevance propagation, which is not making use of the gradient information—and therefore agnostic to the type of neurons in the network, —still produces similar results across different types of neurons. Explanations learned by the rectifying network are arguably more global, because the rectified linear units are prevented from saturating on both sides and therefore more linear. Explanations produced by a rectifying network trained for a shorter time (rect^⋆^) are clearly more global, as evidenced by the positive and negative parts of the pixel-wise decompositions spreading over larger pixel areas.

**Fig 17:**
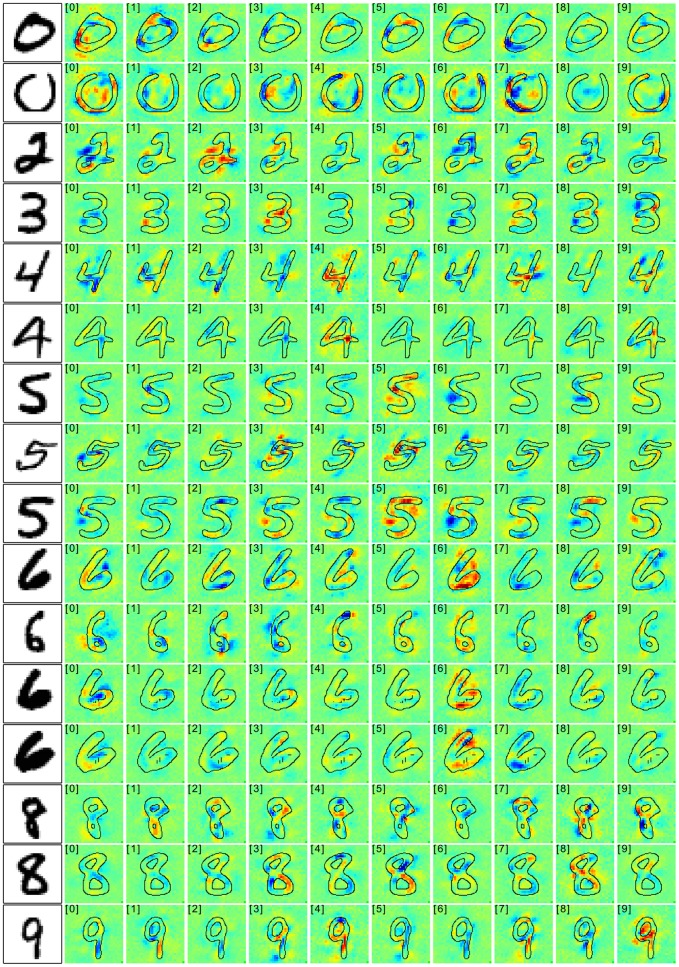
Pixel-wise decompositions for all classes for 16 randomly drawn digits from the MNIST test set. Results are obtained using the relevance propagation Formula (56) with the rectifying network trained for 1 000 000 iterations from Section *MNIST experiments *II**.

In order to compute the pixel-wise decompositions, we used Eq (56), which does not use numerical stabilizers. Use of numerical stabilizers causes relevance to leak from one layer to another, in particular, when the relevance of a particular handwritten digit is measured for another class. Fig 17 shows examples of pixel-wise decompositions for 16 randomly drawn digits sorted according to the digit class.

#### Pixel flipping experiments using the rect-long model from Section *MNIST experiments *II**

In this section we intend to make a semi-quantitative analysis of the pixel-wise decompositions. The basic idea is to compute a decomposition of a digit for a digit class and then flip pixels with highly positive, highly negative scores or pixels with scores close to zero and then to evaluate the impact of these flips onto the prediction scores. The advantage of demonstrating this on MNIST data is that a vast majority of pixels either has very high (black) or very low (white) values, with very few pixels having values in between. For results on photographic scenes one may need to resort to building masks which are specific to each category, for example, a gray box may be good for masking a flower, but it will not mask effectively a gray road or fur of a Koala bear.

In general: If we *flip* a pixel, we do *flipped* = *pixel* ⋅(−1). In further experiments we will consider the digits three and four as inputs. The test set contains 983 fours and 1011 threes, over which the mean prediction is calculated.

**Fig 18:**
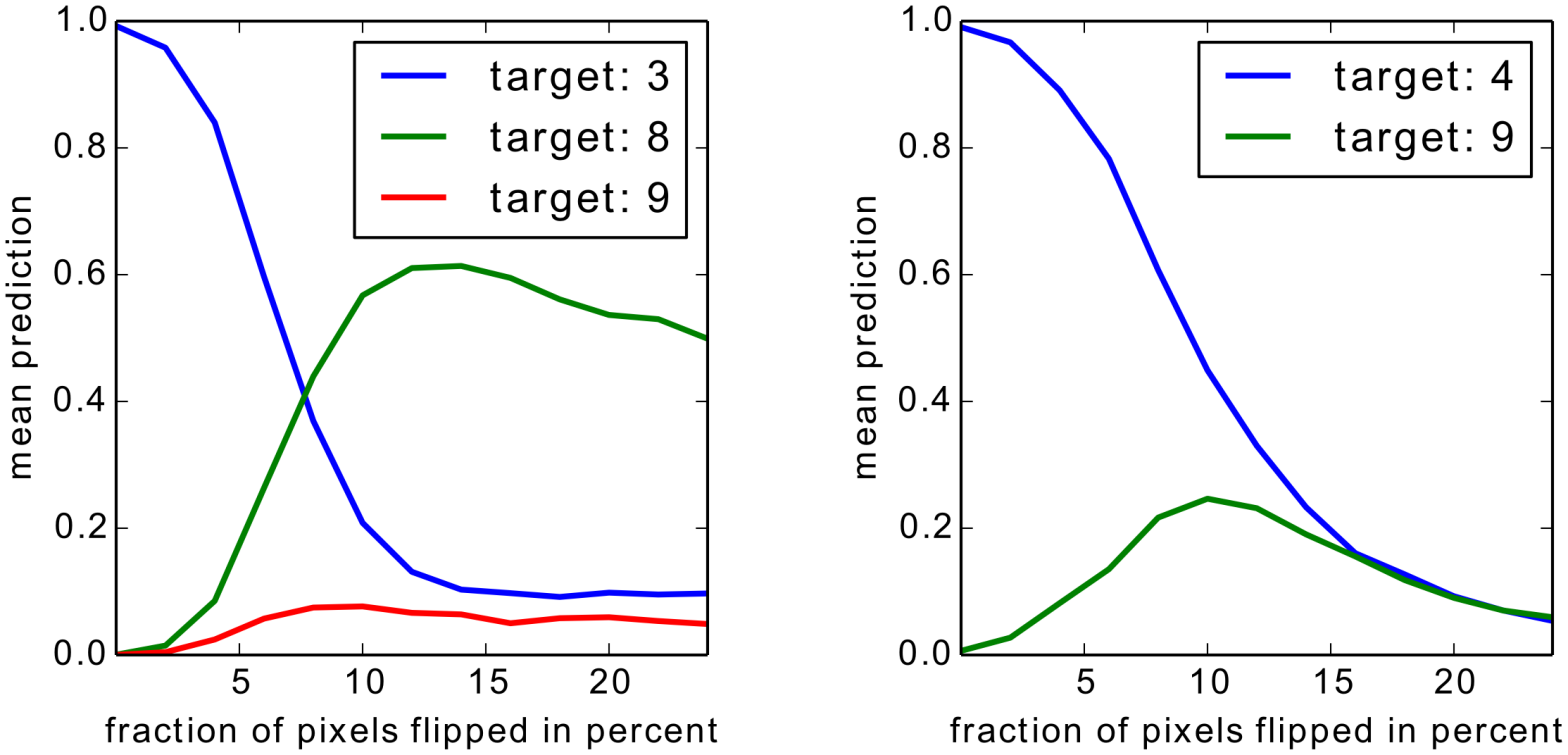
Flipping of high-scoring non-digit pixels. Pixels with highest positive scores are flipped first. The pixel-wise decomposition was computed for the true digit class, three (left) and four (right).

One apparent result from the preceding section is that we can observe non-digit pixels with highly positive pixel scores. In Fig 18 we evaluate the impact of flipping non-digit pixels with highly positive pixel scores on the resulting classifier prediction. We observe in Fig 18 that the classifier prediction for the true digit class, namely three and four, decreases fast when the non-digit pixels with high pixel scores are flipped. This shows, firstly, that having non-digit pixels with high positive pixel scores makes sense for a correct classification. We see also, that flipping the highest scoring pixels for a digit three turns it into a digit eight, which is consistent to the decompositions seen in Fig 16.

Secondly, it shows that measuring the quality of a pixel-wise decomposition by an object segmentation mask is not always a good idea. When seeing the digit as an object, having non-object pixels with high scores can make sense in the case of geometric constraints for objects which are to be recognized, as we have in our case. For this reason we deliberately did not choose object segmentation masks for the digits as a basis for evaluation in the sense that digit-pixels should have highly positive scores, and non-digit pixels should have zero or negative scores.

For the same reason, namely the possibility of the presence of geometric constraints, pixel-wise decomposition is not always a good weak segmentation in contrast to the convincing results in the experiments of [22] on images from the ILSVRC data set. Another reason for a possible divergence between pixel-wise decomposition and segmentation is the possible influence of context in a scene.

**Fig 19:**
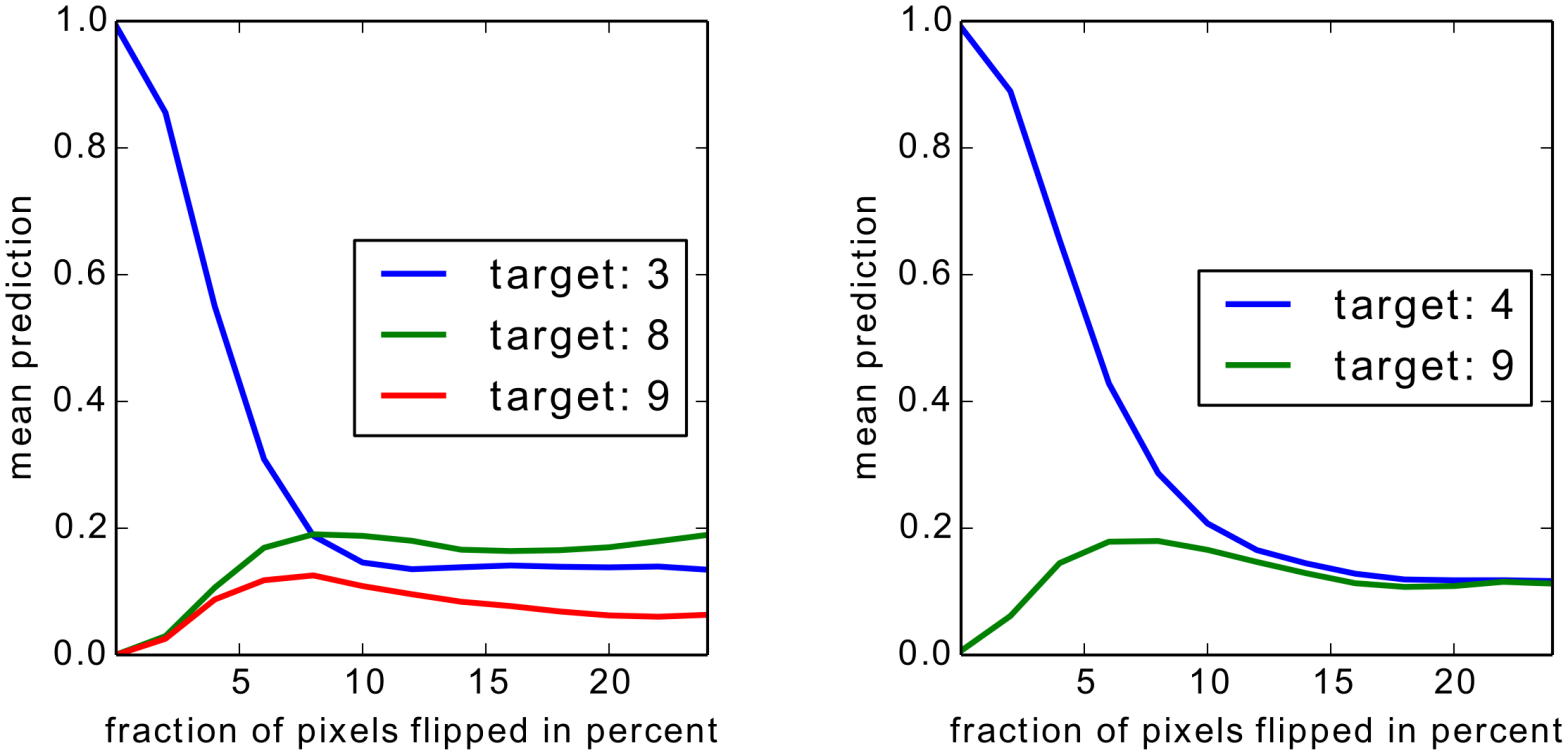
Flipping of digit and non-digit pixels with positive responses. Pixels with highest positive scores are flipped first. The pixel-wise decomposition was computed for the true digit classes three (left) and four (right).

**Fig 20:**
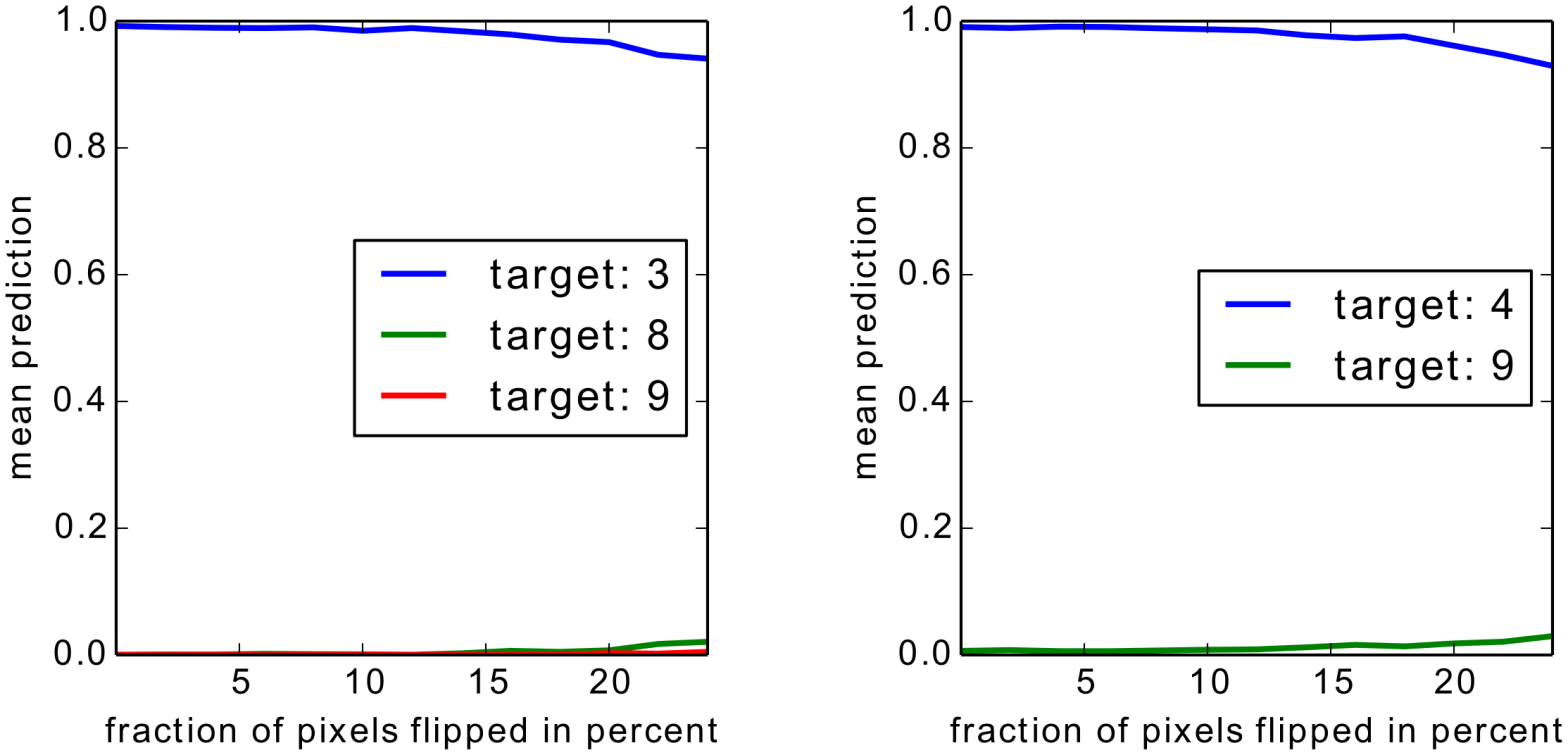
Flipping of pixels with pixel-wise decomposition score close to zero. Pixels with absolute value closest to zero are flipped first. Digit and non-digit pixels may be flipped. Pixel-wise decomposition have been computed for the true digit classes three (left) and four (right).

Once we have established the reason why we do not use segmentation masks for evaluating the quality of pixel-wise prediction we can observe the effects of flipping the highest scoring pixels, independently of whether they are a digit or non-digit pixel. We can see from Fig 19 that the prediction of the correct digit class decreases sharply in this case as well. This decrease can be compared to the case when we flip those pixels whose absolute value of the decomposition score *s* is closest to zero by sorting the pixels according to 1 − ∣*s*∣. We see in Fig 20 that the classifier prediction for the true class decreases only very slowly, thus neutrally scored pixels are less relevant for the prediction. The comparison between Figs 19 and 20 shows that the decomposition score is able to identify those pixels which play little role in the classification of a digit and those pixels which are important for identifying a digit.

Thus, the pixel-wise decomposition is not only intuitively appealing to a human but also makes sense for the representation used in classifier to make its decision. Using digits for demonstrating such a statement has a mild bias towards our method because for a geometric-driven task like digits we can expect that firstly the problem can be learned well by a classifier so that the resulting pixel-wise decompositions are very informative, and secondly what has been learned on digits might be more similar between humans and algorithms compared to complex natural scene recognition tasks. See, however, the experiments in the Section *Neural Network for 1000 ILSVRC classes* on the categories from ILSVRC challenge for results on an object recognition tasks on photographic images which also yield results plausible to a human. In general one can expect that a classifier with poor recognition ability will yield also pixel-wise decompositions with less informative scores.

**Fig 21:**
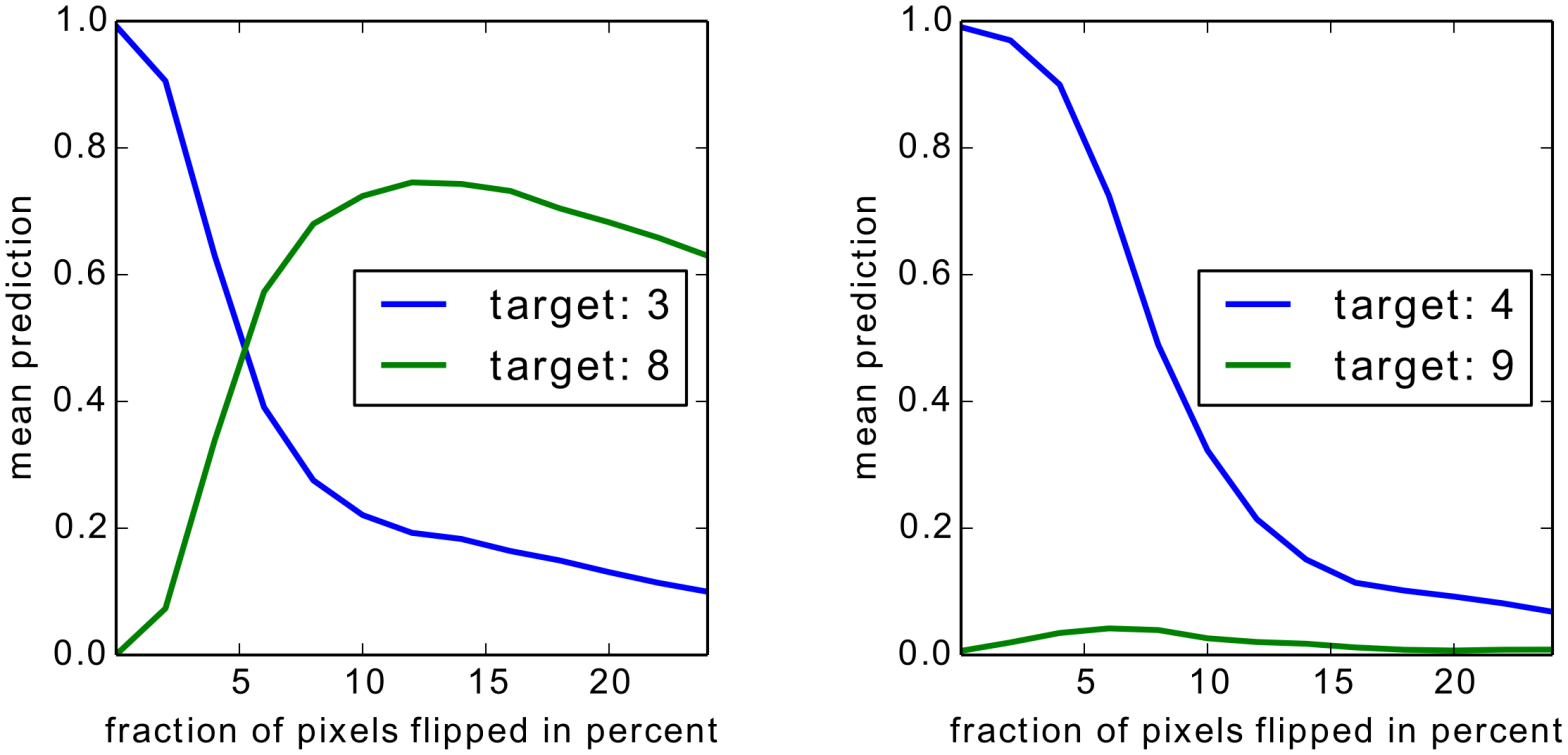
Flipping of pixels with negative responses, due to a pixel-wise decomposition for prediction targets 8 (for digits 3 on the left) and 9 (for digits 4 on the right). Pixels with lowest negative scores are flipped first.

Finally, we evaluate the influence of pixels with negative scores. For this, we take a digit, compute the pixel-wise decomposition for a wrong class, then flip the pixels which are marked negatively when trying to predict the wrong class. Results are shown in Fig 21. We observe for both example cases that the prediction for the true class declines sharply as we tweak the digits towards the wrong class. For digit 9 as target and digits 4 as inputs this flipping results in a noisy blob on top of a stick, such that the result is not cleanly predictable as a nine. However one can observe still a moderate rise of the prediction of the 4 as a 9. For digit 8 as target and digits 3 as inputs we can see, that for some intermediate percentage of flips, the 3 will be identified as an 8. In summary, negatively scored pixels are able to represent what is contradictory to classifying a digit to belong to a particular class.

**Fig 22:**
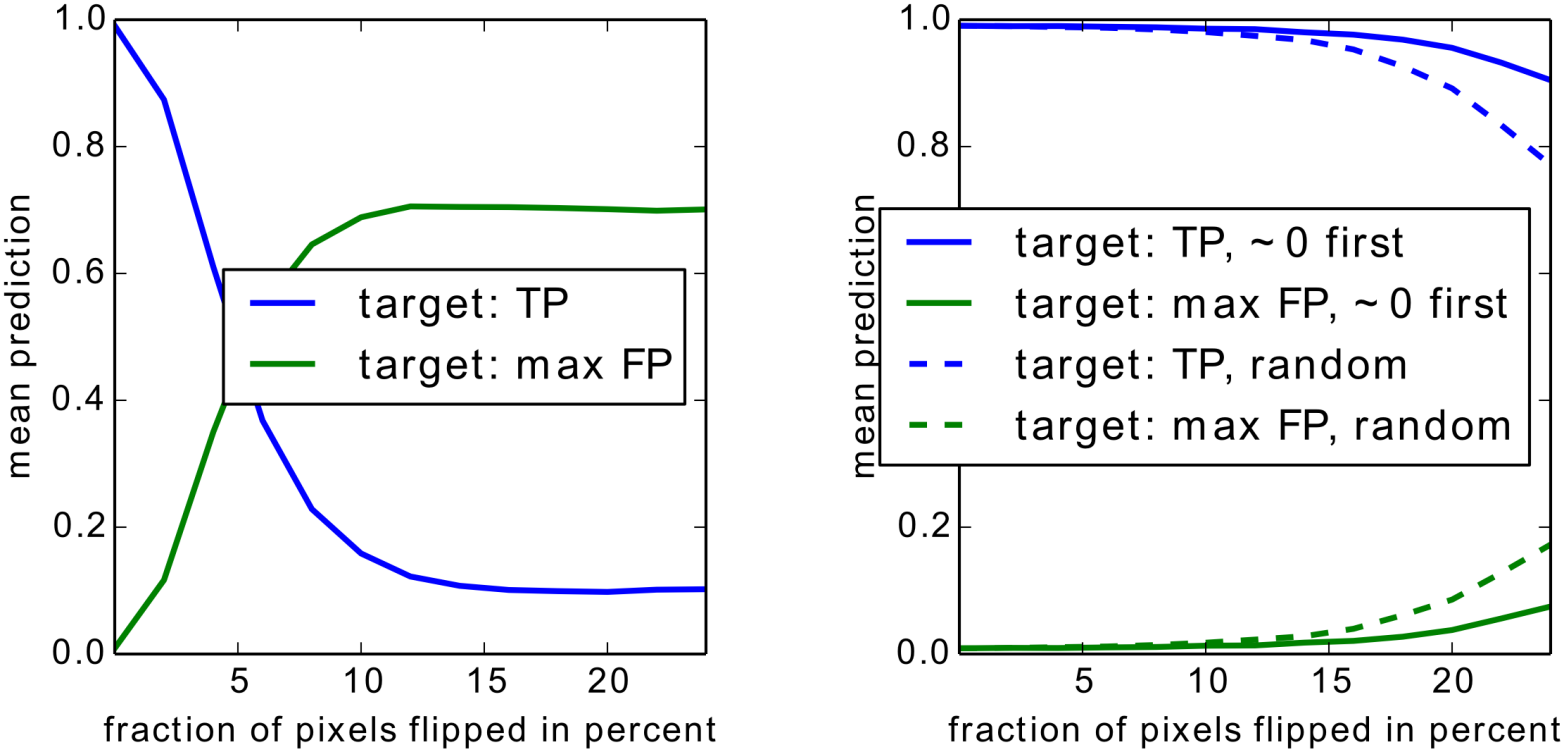
Flipping of pixels for digit and non-digit pixels, compared for each modified digit for the true class against the maximal prediction of all wrong classes. Left: Pixels with highest positive scores are flipped first. Right: Flipping of neutrally predicted pixels, i.e. pixels with absolute value closest to zero are flipped first (solid lines), and flipping of randomly picked pixels (dashed lines). Results are averaged over digits from all digit classes in contrast to using only digit classes 3 and 4 in the preceding figures.

The results in Figs 18, 19 and 20 are shown for the true class against one or more particular wrong classes. The results hold also when we show the behavior of the classifier predictions under flipping of the highest scoring and the most zero-like pixels when we compared for each digit against the maximum of predictions over all wrong classes. Furthermore we compute the average over all 10 digits, not only digits 3 and 4 as true classes as shown in Figs 18, 19 and 20. This result is shown in Fig 22.

**Fig 23:**
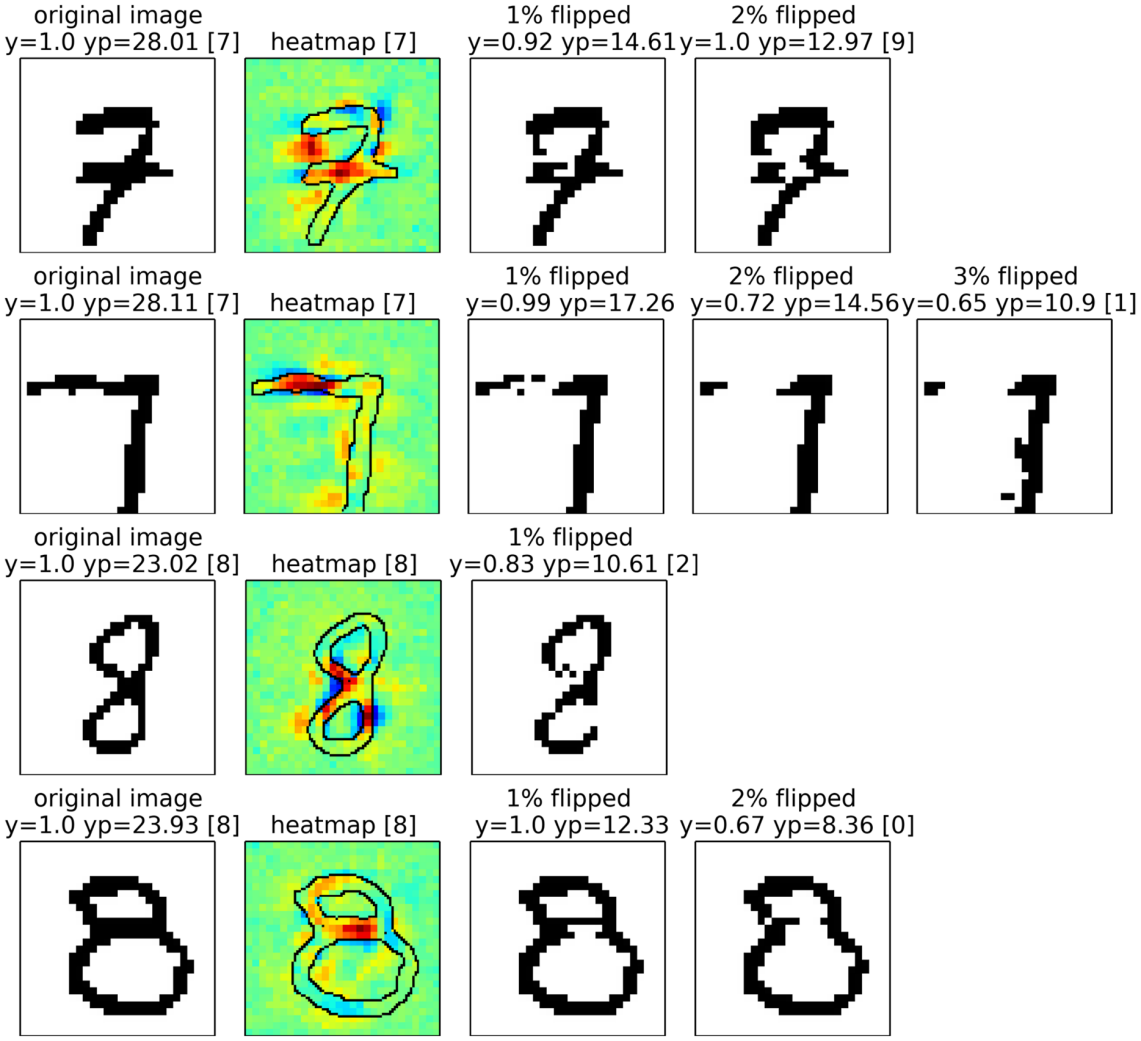
Examples of images with an increasing amount of flipped pixels and the corresponding predictions of the classifier. Here pixels are flipped away from the class label given in parentheses above the heatmap. Pixels were flipped in steps of 1% of all pixels until the predicted class label changed. The plots show the output of the softmax function *y* and the output score of the preceding linear layer *yp*. The pixels were sorted before flipping in *decreasing* order of the pixel-wise score, i.e. highest scoring pixels were flipped first. In this panel the heatmap was computed for the classifier which produced the highest score, i.e. for the predicted class label. The originally predicted label is given on the leftmost image in parentheses, the predicted label after the switch of the prediction is given in the rightmost image.

**Fig 24:**
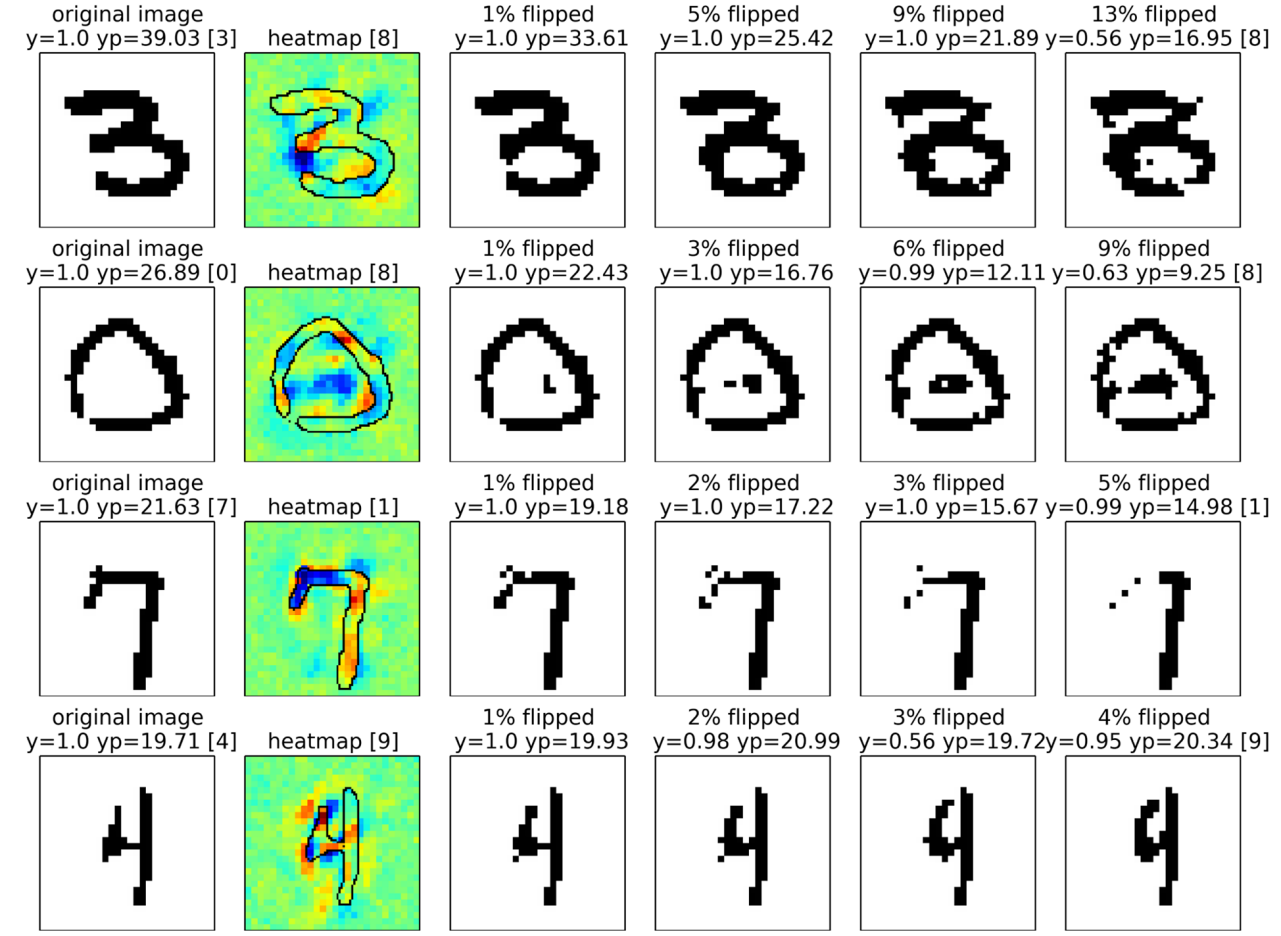
Examples of images with an increasing amount of flipped pixels and the corresponding predictions of the classifier. Here pixels are flipped towards the class label given in parentheses above the heatmap. Pixels were flipped in steps of 1% of all pixels until the predicted class label changed. The plots show the output of the softmax function *y* and the output score of the preceding linear layer *yp*. The pixels were sorted before flipping in *increasing* order of the pixel-wise score, i.e. lowest scoring pixels were flipped first. In this panel the heatmap was computed for a classifier which did not produce the highest score, i.e. for a random false class label. The originally predicted label is given on the leftmost image in parentheses, the predicted label after the switch of the prediction is given in the rightmost image.

On average over all digits, flipping the highest scoring pixels at first results in a fast decline of the prediction for the true class, and at some point another class is predicted. Flipping the pixels at first with scores close to zero results in a much slower decline of the prediction for the true class. This result demonstrates a quantifiable plausibility of the pixel-wise decomposition by layer-wise relevance prediction. In order to visualize the process of pixel flipping, Figs 23 and 24 provide examples of a digit, its heatmap and the resulting images with increasing amounts of flipped pixels. For each of the resulting images the softmax output *y* and the linear layer output *yp* are shown. The first difference between Figs 23 and 24 is that in the former the pixels were flipped according to the heatmap for the highest scoring class, and in the latter the pixels were flipped according to the heatmap for a random class which did not yield the highest score. The second difference is that in Fig 23 the highest scoring pixels were flipped first, which implies that evidence for the predicted class label is removed. As a consequence, the original prediction is gradually lost without giving a specific false class label as target. The digit in Fig 23 changes towards that class label which the classifier and the heatmapping implicitly perceive as the nearest neighbor. With respect to the second difference, in Fig 24 the lowest scoring pixels for the random false class label were flipped first, which implies that evidence against the false class label is removed. As a consequence, the digit is modified towards the specific false class label which is denoted in parentheses above the heatmap. In Fig 23 each of the digits 7 and 8 results into different class labels after flipping. We can see that destroying evidence for a given predicted class may result into differing final class labels. The nearest neighbor of a digit is not constant within the digit class, but depends on the style of the digit itself. Furthermore we can see that pixel-flipping sometimes removes and sometimes adds black pixels, depending on where the highest pixel scores are located. In Fig 24 we can see results of flipping towards a specified false class label. In both cases the resulting digits are plausible to the human observer. Note also small details, e.g. the 4 in Fig 24 is rounded at its lower left end during flipping, while the classifier regards the 7 in Fig 24 to be too short for being a 1 as seen on the blue spot above the vertical bar of the digit. Finally, Fig 17 provides examples of the pixel-wise decomposition for 16 randomly drawn digits and the predictors for all 10 digit classes.

### Neural Network for 1000 ILSVRC classes

**Fig 25:**
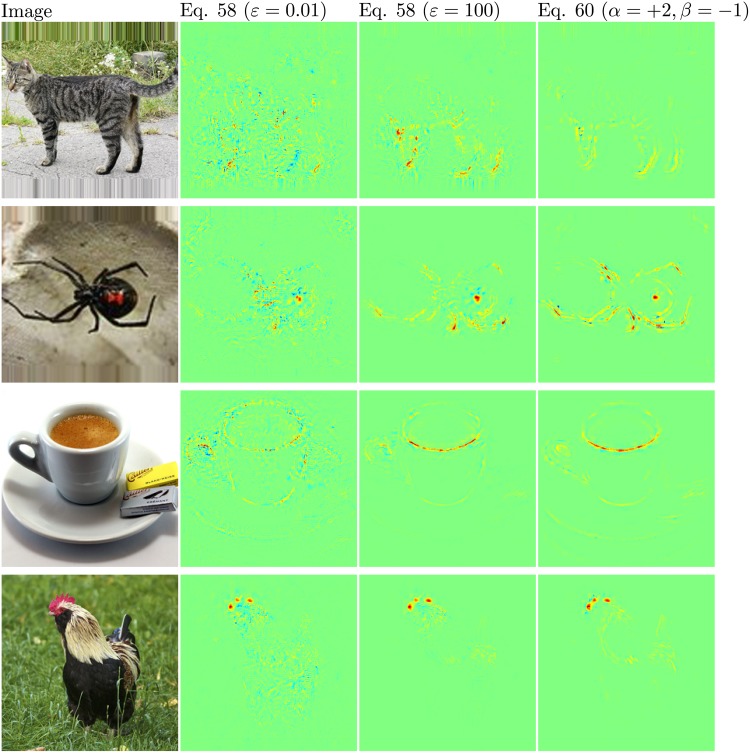
The pixel-wise decompositions for examples images of the neural net pre-trained on ILSVRC data set images and provided by the Caffe open source package [60]. Second column shows decompositions computed by Formula (58) with stabilizers *ɛ* = 0.01, the third column with stabilizers *ɛ* = 100, the fourth column was computed by Formula (60) using *α* = +2, *β* = −1. The artifacts at the edges of the images are caused by filling the image with locally constant values which comes from the requirement to input square sub-parts of images into the neural net. Pictures in order of appearance from Wikimedia Commons by authors Jens Nietschmann, Shenrich91, Sandstein, Jörg Hempel.

We use here the pre-trained neural network which is provided by the Caffe open source package [60]. Fig 25 shows pixel-wise decompositions for example images from categories of the ILSVRC data set. Results are shown on images from Wikimedia Commons rather than the ILSVRC images in order to avoid license problems when showing images. Unsurprisingly the evidence is not as sharp as for the MNIST data set because the underlying classification problem is much more diverse with a higher complexity of classes and images to be processed. We can see for the rooster high scores at his cockscomb and parts of his feathers. The black widow spider has high evidence in her head section which is surprising because it is not so prominent to humans but is consistent among other images of black widow spiders as well. Further high evidence lies in parts of black widow legs, and, as expected, the characteristic red spots of a black widow. For the tabby cat we see evidence in his fur but also in the road surface under the cat which has similar color and texture to a cat’s fur. The espresso cup has high responses on the cup’s edges, the grip, some evidence in the espresso liquid, however only little response for the edges on the accompanying sweets which has the wrong color, gray, for an espresso. Note that a good classifier for a concept does not necessarily imply that an object can be localized very well by its pixel-wise decomposition. Since the classifier processes an image globally, it can perceive evidence away from the actual object, for example by taking into account typical background of objects, the context of an object or global statistics which are correlated to an object. In that sense, for example, bottles can serve as evidence for a dining table or sky can be a clue for photos with airplanes on the ground or photos of outside views of churches.

**Fig 26:**
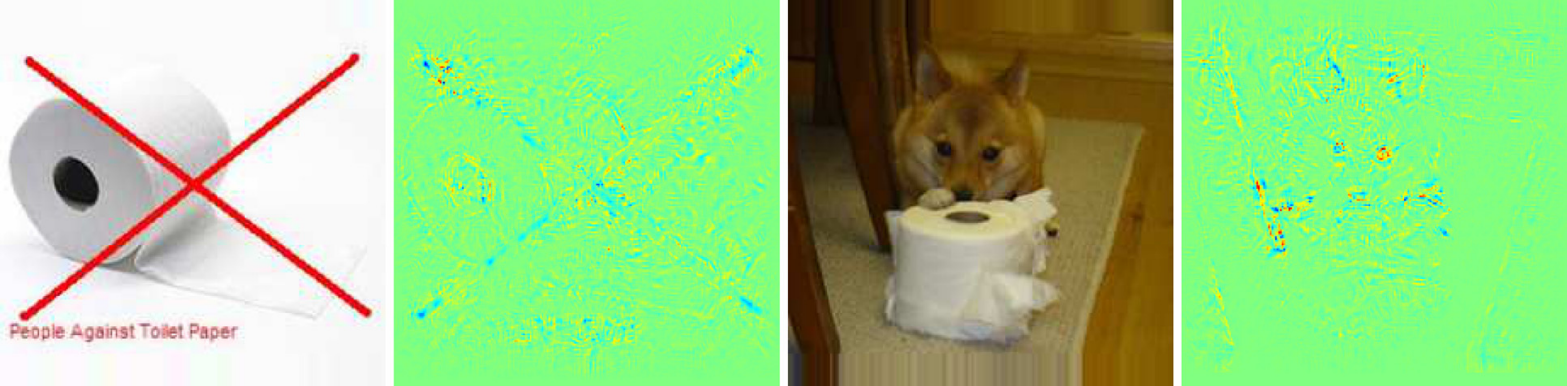
Failure examples for the pixel-wise decomposition. Left and Right: Failures to recognize toilet paper. The decompositions computed by Formula (58) with stabilizers *ɛ* = 0.01 The neural net is the pre-trained one on ILSVRC data from the Caffe package [60]. The computing methods for each column are the same as in Fig 25. Pictures in order of appearance from Wikimedia Commons by authors Robinhood of the Burger World and Taro the Shiba Inu.

Fig 26 shows examples where the pixel-wise decomposition does not yield discriminative results. For the toilet paper, positive prediction scores were already low in both examples. This corresponds to our expectation that a less discriminative classifier results also in less convincing pixel-wise decompositions.

**Fig 27:**
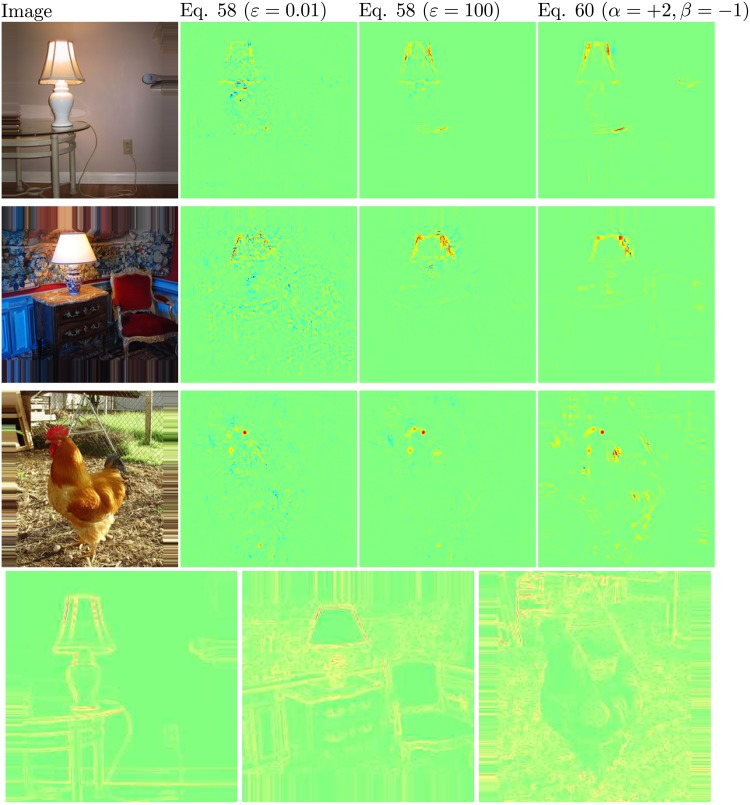
The pixel-wise decomposition is different from an edge or texture detector. Only a subset of strong edges and textures receive high scores. Panels show the original image on the left, and the decomposition on the right. The decompositions were computed twice for the classes table lamp and once for the class rooster. The neural net is the pre-trained one on ILSVRC data from the Caffe package [60]. The computing methods for each column are the same as in Fig 25. The last row shows the gradient norms normalized to lie in [0, 1] mapped by the same color scheme as for the heatmaps. Pictures in order of appearance from Wikimedia Commons by authors Wtshymanski, Serge Ninanne and Immanuel Clio.

Comparing the different shown methods in Figs 25 and 27, one can see that the method (58) with a small value of the stabilizer *ϵ* produces results with the finest granularity and also with the highest amount of negative evidence. When increasing the values of the stabilizer *ϵ*, the method becomes less granular and shows less negative evidence. This result was also observed when comparing other small and large values of the stabilizer *ϵ*. The decrease of negative pixel-wise scores and of granularity can be both explained under some assumptions. Firstly, note that compared to Eq (58) with stabilizer equal to 0 the Eq (58) with stabilizer *ϵ* differs by a multiplicative factor of $\frac{|z_{j}|}{|z_{j}|+\epsilon }$ which goes to zero when ∣*z*_*j*_∣ goes to zero. Thus dampening by this factor increases when the absolute value of inputs ∣*z*_*j*_∣ decreases. Secondly note, that in Eq (58) the sign of the relevance *R*_*j*_ of input neuron *j* will be flipped in the messages $R_{i\leftarrow j}^{\left(l,l+1\right)}$ for either the positive or the negative inputs *z*_*ij*_ (depending on the sign of $\frac{z_{ij}}{z_{j}}$). When we assume that the neurons at layer *l* + 1 with small absolute value of inputs ∣*z*_*j*_∣ are responsible for generating a high proportion of all negative messages $R_{i\leftarrow j}^{\left(l,l+1\right)}<0$, then the reduction of negative pixel-wise scores by increasing stabilizer *ϵ* can be explained. Such an assumption can be reasonable because a small absolute value of inputs can sometimes be explained by the sum of positive inputs being close to the sum negative inputs, and noting that either the positive or the negative sets will produce negative messages $R_{i\leftarrow j}^{\left(l,l+1\right)}<0$. Therefore, in case of small inputs, sometimes a large fraction of the relevance *R*_*j*_ can be turned into negative messages. On the other side, one can expect that in a neural network which predicts a positive score *f*(*x*) > 0, a majority of network inputs is positive, and a majority of neuron relevances *R*_*j*_ is positive, since their sum within a layer equals *f*(*x*) > 0. In particular one may expect from that, that the majority of neurons with large inputs *z*_*j*_ have positive relevances *R*_*j*_ > 0, and the messages for neurons with large inputs *z*_*j*_ are mostly positive. In conclusion, positive scores are less affected by dampening when *f*(*x*) > 0. Canceling out negative scores by a large value of *ϵ* in intermediate layers leads also to more positive scores observed in the final heatmaps, and thus also to less visual granularity. We emphasize that this is a qualitative argument which we validated by checking a small number images rather than a universally holding claim. As for properties of Eq (60), we note that a choice of *β* < 0 such that *α* = 1 − *β* fixes the ratio of negative to positive messages to $\frac{-\beta }{1-\beta }$ for each neuron. In particular, the sum of the negative messages is upper bounded by the sum of the positive messages. For rectified linear neurons this is a reasonable assumption because they fire only when their input is positive. In conclusion, our choice *β* = −1, yielding a ratio of 1:2 towards positive messages explains why the heatmaps for the method (60) in Figs 25 and 27 are dominantly positive.

It is known from [21] that lower layers of deep networks which are very similar to the one from [60] used here may act like learned texture or gradient detectors. Fig 27 shows that the pixel-wise decomposition is, however, not equivalent to simply assigning scores to dominant edges. The original images from Fig 27 have many regions with strong gradients, however the pixel-wise decomposition assigns notable scores only to a small fraction of pixels with large gradients. Note for example that the highly textured and visually prominent wallpaper in the middle example with the table lamp receives only small scores. The same holds for the artifacts at the edges of the images which come from filling the image with locally constant values which is a compromise in order to make these images to be a square.

## Discussion

Nonlinear learning machines are ubiquitous in modern problem solving. While highly successful in e.g. hard classification, regression or ranking problems, their non-linearity so far has prevented these models to additionally explain and thus contribute to a better understanding about the nature of the solved problem. Making, say, a nonlinear classification decision for one particular novel data point transparent to the user is essentially orthogonal to the standard task of optimizing for an excellent and well generalizing classifier. We have introduced a tool set for deconstructing a nonlinear decision and thus fostering transparency for the user.

In particular we have introduced the general concept of decomposition of a nonlinear image classification decision in terms of pixels. In other words, for a well-classified image, a heatmap can be produced that highlights pixels that are responsible for the predicted class membership. Note that this is possible without need of segmented training images. We consider heatmapping as an important part of the interpretation of nonlinear learning machines and its applicability goes far beyond what has been exemplarily presented in this work: it ranges from the interpretation of bio-medical images to the practical validation of a trained models for image classification.

Practically, we have proposed two different approaches to pixel-wise decomposition: The first one, Taylor-type decomposition, seeks to linearly approximate the class scoring function locally by performing a Taylor decomposition of it near a neutral data point without class membership, where the contribution of each dimension (i.e. pixel) can easily be identified. The second one, coined layer-wise relevance propagation, applies a propagation rule that distributes class relevance found at a given layer onto the previous layer. The layer-wise propagation rule was applied iteratively from the output back to the input, thus, forming another possible pixel-wise decomposition. This inherits the favorable scaling properties of backpropagation.

Notably, these two methods were not defined as a particular solution to the heatmapping problem, but instead as a set of constraints that the heatmapping procedure must fulfill in order to be admissible. For instance, the exact choice of Taylor reference point was not specified beyond the constraints that it should be a root and that it should be located close to the actual data point. Similarly, the layer-wise relevance propagation has been defined with the sole restriction that the propagation rule conserves class relevance on a layer and node basis. Thus, the further specification of relevance propagation rule is deferred to the appreciation of the user, or as a future work, and may either be model-specific, problem-specific, or subject to a particular practical or computational requirement.

Specific instances of the pixel-wise decomposition procedure satisfying the constraints mentioned above have been proposed and analyzed. In particular, our work has covered a set of non-linear learning algorithms for image classification, including kernel classifiers over Bag of Words pooled features, and feed-forward multilayer neural networks. Both models are popular choices for image classification or analysis.

Our experiments show that applying Taylor-type decomposition, layer-wise relevance propagation or a combination of both on these non-linear models produces highly informed heatmaps that reflect in many aspects the sophistication of the learned classifier. In particular, we have demonstrated that the same relevance propagation rule may, for different images, react to a variety of image features within the bounds modeling capacity. For example, in the case of the ImageNet convolutional network, we have shown that the heatmapping procedure finds class-relevant features that can be large areas of a particular color, localized features, image gradients, or more structured visual features such as edges, corners, contours, or object parts.

An important aspect of the proposed heatmapping procedure lies in the fact that it does not require to modify the learning algorithm, or to learn an additional model for heatmapping. Instead, it can be directly and transparently applied to any (pre-)trained Bag of Words model or neural network when applicable. This desirable property is demonstrated in this paper by the heatmapping of images classified by the third-party GPU-trained ImageNet neural network. In particular, our heatmapping procedure was applied to this network without any further training or retraining. Thus, heatmaps for the ImageNet network could be quickly produced using a modest CPU.

While we have proposed in this paper several instances of pixel-wise decomposition and demonstrated their excellent performance in practice, the set of possible relevance propagation methods, and their mathematical properties, will certainly have to be further explored. A first aspect that needs to be investigated is the greediness of the layer-wise relevance propagation procedure, in the sense that it is computed one layer at a time, and its potential impact on the quality of heatmaps: While computationally advantageous, some of the backpropagated relevance might encounter a dead-end in the lower layers and be distributed randomly. Another open question relates to the heuristic nature of the proposed instances of relevance propagation, in particular, whether the distributed relevance messages being proportional to the weighted neuron activations can be analytically justified.

Finally, it is not clear how to evaluate the quality of a heatmap beyond simple visual assessment. In this paper we have proposed as a starting point a pixel-flipping method that allows to discriminate between two heatmapping methods that may otherwise look of similar quality to the human. Finding quantitative properties that are desirable for these heatmaps, or further constraints on the heatmapping procedure is therefore of nature to complement the human assessment. Future work will need to explore the many domain- and data-specific degrees of freedom in the heatmapping process in order to ultimately propose a universal metric for quantification. It is our firm belief, that heatmapping will be an important ingredient of future knowledge discovery and exploratory analysis and understanding of complex data in the sciences and industry, even beyond the presented field of image analysis.
